# Signal peptide peptidase activity connects the unfolded protein response to plant defense suppression by *Ustilago maydis*

**DOI:** 10.1371/journal.ppat.1007734

**Published:** 2019-04-18

**Authors:** Niko Pinter, Christina Andrea Hach, Martin Hampel, Dmitrij Rekhter, Krzysztof Zienkiewicz, Ivo Feussner, Anja Poehlein, Rolf Daniel, Florian Finkernagel, Kai Heimel

**Affiliations:** 1 Department of Molecular Microbiology and Genetics, Institute of Microbiology and Genetics, Göttingen Center for Molecular Biosciences (GZMB), University of Göttingen, Göttingen, Germany; 2 Department of Plant Biochemistry, Albrecht-von-Haller-Institute for Plant Sciences, Göttingen Center for Molecular Biosciences (GZMB), University of Göttingen, Göttingen, Germany; 3 Service Unit for Metabolomics and Lipidomics, Göttingen Center for Molecular Biosciences (GZMB), University of Göttingen, Göttingen, Germany; 4 Department of Genomic and Applied Microbiology & Göttingen Genomics Laboratory, Institute of Microbiology and Genetics, University of Göttingen, Göttingen, Germany; 5 Center for Tumor Biology and Immunology (ZTI), Institute of Molecular Biology and Tumor Research (IMT), Marburg, Germany; Institute of Microbiology, CHINA

## Abstract

The corn smut fungus *Ustilago maydis* requires the unfolded protein response (UPR) to maintain homeostasis of the endoplasmic reticulum (ER) during the biotrophic interaction with its host plant *Zea mays* (maize). Crosstalk between the UPR and pathways controlling pathogenic development is mediated by protein-protein interactions between the UPR regulator Cib1 and the developmental regulator Clp1. Cib1/Clp1 complex formation results in mutual modification of the connected regulatory networks thereby aligning fungal proliferation *in planta*, efficient effector secretion with increased ER stress tolerance and long-term UPR activation *in planta*. Here we address UPR-dependent gene expression and its modulation by Clp1 using combinatorial RNAseq/ChIPseq analyses. We show that increased ER stress resistance is connected to Clp1-dependent alterations of Cib1 phosphorylation, protein stability and UPR gene expression. Importantly, we identify by deletion screening of UPR core genes the signal peptide peptidase Spp1 as a novel key factor that is required for establishing a compatible biotrophic interaction between *U*. *maydis* and its host plant maize. Spp1 is dispensable for ER stress resistance and vegetative growth but requires catalytic activity to interfere with the plant defense, revealing a novel virulence specific function for signal peptide peptidases in a biotrophic fungal/plant interaction.

## Author summary

Biotrophic pathogens establish compatible interactions with their host to cause disease.

A critical step in this process is the suppression of plant defense responses by secreted effector proteins. In the maize infecting fungus *Ustilago maydis* expression of effector encoding genes is coordinately upregulated at defined stages of pathogenic development in so-called effector waves. Efficient secretion of the multitude of effectors relies on the unfolded protein response (UPR) to maintain homeostasis of the endoplasmic reticulum. Activation of the UPR is connected to the control of fungal proliferation through direct protein-protein interactions between the UPR regulator Cib1 and the developmental regulator Clp1. Here, we show that this interaction leads to functional modification of Cib1 and modulation of UPR gene expression to adapt the UPR for long-term activity in the plant. Within a core set of UPR regulated genes we identify the signal peptide peptidase Spp1 as a key factor for fungal virulence. We show that Spp1 requires its conserved catalytic activity to suppress the plant defense and cause disease. The virulence specific function of Spp1 does not involve pathways previously known to be associated with Spp1-like proteins or plant defense suppression, suggesting a novel role for Spp1 substrates in biotrophic interactions.

## Introduction

Microbial cells modulate conserved signaling pathways to adjust their intracellular physiology to constantly changing environmental conditions [1–3]. The smut fungus *Ustilago maydis* is a highly adapted biotrophic pathogen of its host plant maize that establishes a compatible fungal/plant interaction without evoking obvious plant defense responses [4]. The switch from saprophytic to biotrophic growth of *U*. *maydis* is initiated after fusion of two compatible haploid sporidia, generating an infectious filamentous dikaryon that strictly depends on living host tissue for further propagation [5]. Filaments elongate by tip-growth on the leaf surface and do not actively divide, as their cell cycle is arrested [6, 7]. After appressoria-mediated plant penetration, the cell cycle arrest is released and mitotic growth of the dikaryotic filament initiated, followed by massive proliferation of dikaryotic hyphae and tumor formation [8]. The central regulator of pathogenic development in *U*. *maydis* is the bE/bW transcription factor complex, encoded by the *b*-mating type locus [9, 10]. The *b*-dependent transcriptional network comprises 345 genes and controls the early steps of pathogenic development including filament formation, maintenance of the G2-cell cycle arrest, appressoria formation and plant penetration [11, 12]. Further development *in planta*, however, requires modulation of the *b*-dependent regulatory network by the Clp1 protein, mediating cell cycle release and mitotic proliferation *in planta* [8].

To cope with the plant immune system and establish a compatible interaction, biotrophic pathogens secrete effector proteins that suppress the plant defense and reprogram host metabolism [13, 14]. In *U*. *maydis*, the apoplastic effectors Pep1 and Pit2 actively interfere with the plant defense response by inhibiting the host peroxidase POX12, and cysteine proteases, respectively [15–18]. In addition, Tin2 and the chorismate mutase Cmu1 manipulate the plant host metabolism to foster colonization by *U*. *maydis* [19, 20]. Effector encoding genes are expressed in an organ and tissue specific manner and are coordinately upregulated during biotrophic growth resulting in effector waves [21, 22] that impose enormous stress on the protein folding machinery in the endoplasmic reticulum (ER). In the budding yeast *Saccharomyces cerevisiae*, ER stress is sensed by the ER-membrane localized kinase/RNase Ire1, leading to Ire1 activation and endonucleolytic cleavage of the constitutively expressed *HAC1* mRNA. The processed mRNA encodes the bZIP transcription factor Hac1 that is functionally conserved in most eukaryotes including mammals (Xbp1) and *U*. *maydis* (Cib1) [23, 24]. Elevated expression of Hac1 target genes promotes the restructuring of the secretory pathway by increasing ER folding capacity, ER expansion and degradation of irreversibly misfolded proteins via the ER associated degradation (ERAD) pathway [25–27]. One mechanism to target proteins for ERAD is mediated by signal peptide peptidases (SPPs) which are ER membrane-localized aspartyl-proteases that cleave type II oriented transmembrane proteins and remnant signal peptides [28–31]. Although conserved in their catalytic activity, SPPs exert diverse physiological roles including antigen representation in human cells [32], transcription factor activation during hypoxia in *Aspergillus* species [33] or regulation of embryonic development in *Caenorhabditis elegans* [34].

A functional UPR is crucial for virulence in various human and plant-pathogenic fungi, including *U*. *maydis* [35–41] and important for efficient secretion of hydrolytic enzymes involved in decomposition of complex polysaccharides including cellulose and plant cell walls in the fungal saprophytes *Trichoderma reesei* and *Neurospora crassa* [42–45]. In *U*. *maydis*, the UPR is connected to the regulation of biotrophic development and specifically activated after plant penetration. Expression of the UPR regulator Cib1 and its interaction with the developmental regulator Clp1 leads to accumulation of Clp1 protein and triggers re-initiation of mitotic growth *in planta* [36]. Moreover, Cib1 is required for efficient secretion of effector proteins and participates as well in their transcriptional regulation [46]. Apparently, UPR gene expression is adapted to the lifestyle of the fungus and altered by the interaction between Cib1 and Clp1. Both proteins are constantly expressed during biotrophic growth *in planta* [8, 11, 36], and their interaction renders *U*. *maydis* hyper-resistant towards ER stress. It is conceivable that this enables long-term UPR activity *in planta*, which can be otherwise deleterious [36]. Although initial data suggests that this is connected with dampening of UPR activity, the detailed consequences on UPR gene expression have not been addressed, yet.

In addition to the central role of Hac1-like proteins in fungal pathogens, only few UPR regulated genes have been identified that contribute to fungal virulence. Importantly, none of these factors exerts virulence specific functions. In strains lacking the ER chaperone Lhs1 in the hemibiotrophic blast fungus *Magnaporthe oryzae* (*Pyricularia oryzae*), the ER co-chaperone Dnj1 in *U*. *maydis* or the protein disulfide isomerase Pdi1 in the necrotrophic plant pathogen *Botrytis cinerea*, reduced virulence is invariably connected to erroneous protein folding and reduced ER stress resistance [47–49]. Consequently, UPR regulated factors with virulence functions unrelated to ER stress resistance remain to be discovered.

Here, we show that modulation of UPR gene expression by Clp1 correlates with Cib1-phosphorylation and stabilization. We identify the UPR-regulated signal peptide peptidase Spp1 as a novel key virulence factor that is dispensable for vegetative growth or filament formation but is of crucial importance to establish a compatible biotrophic interaction and cause disease. Importantly, the loss of virulence cannot be attributed to altered protein secretion, defects in ER stress resistance or ERAD-mediated protein degradation, but is connected to the catalytic activity of Spp1 itself to interfere with plant defense responses.

## Results

### Cib1 controls expression of a core set of UPR regulated genes

**Fig 1:**
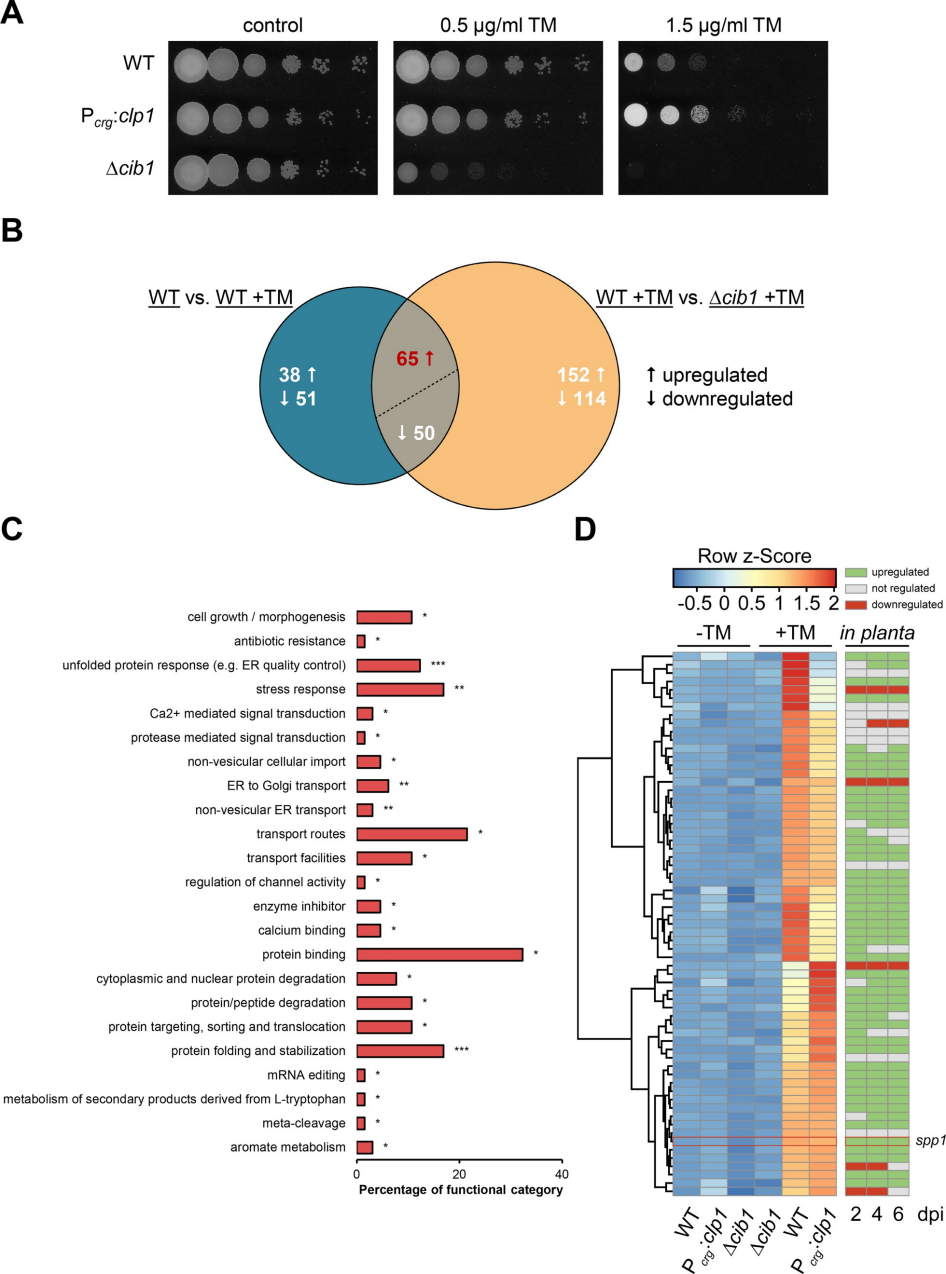
RNAseq analysis identifies a core set of UPR regulated genes. **(A)** ER stress assay of *U*. *maydis* strain JB1 (WT), JB1Δ*cib1* and UVO151 (P_*crg*_:*clp1*). Serial 10-fold dilutions were spotted on YNB solid medium supplemented with 1% arabinose (YNBA) and indicated concentrations of tunicamycin (TM). Plates were incubated for 48 h at 28°C. **(B)** Venn diagram of differentially expressed genes identified by RNAseq analysis. Numbers depict differentially regulated genes (log2FC≥2) in WT +TM compared to the untreated control (blue circle) and in WT +TM compared to the Δ*cib1* +TM control (yellow circle). Both sets share 115 genes (overlap), of which 65 were upregulated and 50 downregulated in a *cib1*-dependent manner. Upward arrows indicate upregulation, downward arrows indicate downregulation. **(C)** Enriched functional categories of the 65 UPR core genes identified by RNAseq analysis. FunCat analysis was performed using the “Functional Catalogue” (FunCat) of the MIPS *U*. *maydis* database (http://mips.helmholtz-muenchen.de/funcatDB/). Bars reflect the percentage of core genes belonging to the respective functional category. Please note that individual genes can be assigned to more than one category. Only overrepresented categories are shown. *P value ≤ 0.05, **P value ≤ 0.01 and ***P value ≤ 0.001. **(D)** Heat map showing expression levels of 65 upregulated genes as determined by RNAseq analysis. Expression values were log2-transformed, mean-centered and SD-scaled reads per million (RPM) (Row z-Score) were calculated and visualized using the ClustVis tool (https://biit.cs.ut.ee/clustvis/). Blue and red color intensity of the row z-Score indicates downregulation and upregulation, respectively. Hierarchical clustering on the y-axis groups genes with similar expression profiles. Euclidean distance and complete linkage for genes were used as clustering method. Sample types (WT, P_*crg*_:*clp1*, Δ*cib1*) and treatment conditions (without tunicamycin [-TM], with tunicamycin [+TM]) are indicated at the bottom. Regulation of the 65 UPR core genes *in planta* [22] is depicted for 2, 4 and 6 days post inoculation (dpi). Green bars indicate upregulation, grey indicate the absence of differential gene expression and red bars indicate downregulation.

Previously, we have demonstrated that the physical interaction between Cib1 and Clp1 leads to drastically increased ER stress resistance (Fig 1A), by preventing deleterious UPR hyperactivation and adaptation of the UPR for long-term activity during biotrophic development of *U*. *maydis* [36]. To identify UPR regulated genes and address their modulation by Clp1 on a global scale we performed RNAseq analysis of *U*. *maydis* under non-stress and tunicamycin (TM)-mediated ER stress conditions. Tunicamycin inhibits N-glycosylation of proteins, leading to accumulation of misfolded proteins in the ER and induction of ER stress. To avoid crosstalk with the *b*-dependent signaling cascade we used *U*. *maydis* strain JB1 (WT), in which the *b*-mating type locus is deleted [11]. The JB1 derivative UVO151 allows for arabinose-inducible expression of *clp1* by the *crg1*-promoter (P_*crg*_:*clp1*) [11] and strain JB1Δ*cib1* (Δ*cib1*) [36] was used as additional control to identify TM-induced side effects unrelated to UPR signaling.

The experiment was performed with three biological replicates for each strain and condition (n = 3). Genes were considered UPR regulated when expression was induced in response to TM-mediated ER stress and no alteration in gene expression was observed in the Δ*cib1* control strain. Differentially expressed genes were filtered at a 4-fold cut-off (log2FC ≥2) and a false discovery rate (fdr) of <0.05. Using these criteria, we identified 204 genes differentially expressed in response to TM treatment (103 upregulated, 101 downregulated) in WT (JB1) background (WT vs. WT +TM). 115 genes of the 204 TM-regulated genes required *cib1* for differential expression and were not affected by TM in the Δ*cib1* control (WT +TM vs. Δ*cib1* +TM). Of these 115 genes, 65 were induced and 50 were repressed in response to TM treatment (Fig 1B and S1 Table).

Since all Hac1-like UPR regulators are transcriptional activators, Cib1-dependent expression of the 50 UPR-repressed genes is likely to be indirect or affected by additional regulators. We thus defined the 65 upregulated as UPR core genes and focused on them in our subsequent analyses. In this set of genes we identified *bip1*, *lhs1*, *mpd1* and *dnj1*, all of which have been identified as UPR regulated genes in previous studies [36, 49]. Under less stringent filtering criteria (log2FC ≥1) 269 UPR core genes were identified, that include *cib1*, *ost3*, *cne1 and pit1* in addition to the previously mentioned genes [36, 46] (S1 Table).

UPR core genes were subjected to enrichment analysis of functional categories using the MIPS functional catalogue database (http://mips.helmholtz-muenchen.de/funcatDB/) (S2 Table).

As expected, functional categories significantly overrepresented within the 65 UPR core genes are associated with the UPR (total of 23 categories), including "protein folding and stabilization" (p = 1.02E^-08^), "unfolded protein response" (p = 2.15E^-07^), "non-vesicular ER transport" (p = 3.87E^-03^), "ER to Golgi transport" (p = 4.85E^-03^), "stress response" (p = 8.57E^-03^), "transport routes" (p = 1.12E^-02^) and "protein binding" (p = 1.19E^-02^) (Fig 1C and S2 Table). Using GeneOntology (GO) analysis (www.geneontolgy.org) 37 of the 65 UPR core genes were functionally classified, revealing significant enrichment only for the GO term GO:0034976 "response to endoplasmic reticulum stress” (p = 1.54E^-03^). Consistently, UPR core genes encode proteins with conserved functions in ER protein folding (Bip1/UMAG_15034, Lhs1/UMAG_00904, Dnj1/UMAG_05173, Dnj2/UMAG_10099, Mpd1/UMAG_05352, Pdi1/UMAG_10156, Ero1/UMAG_05219, Dnj1/UMAG_10099), ER-associated calcium transport (Ena5/UMAG_00204; Pmr1/UMAG_20218), ER-associated degradation (ERAD) (Hrd1/UMAG_00542, Hrd3/UMAG_04355; Der1/UMAG_05898), protein secretion and protein transport processes (Sec11/UMAG_00481, SPC3/UMAG_15029, Sec63/UMAG_06175). No significant enrichment of functional categories was observed for the 50 UPR repressed genes.

Since the UPR is specifically induced after plant penetration (2 days post inoculation (dpi)) and remains constitutively active during the fungal/plant interaction, we compared our UPR core gene set with the genes differentially expressed during biotrophic development (2, 4 and 6 dpi) [22]. This revealed that 51 of the 65 core genes were consistently upregulated by the UPR and during biotrophic development. Interestingly, 6 genes were UPR-induced but repressed *in planta* (Fig 1D) (S3 Table), suggesting that expression of these genes is not solely regulated by the UPR and is most likely affected by other, potentially plant-related cues. By contrast, of the 50 UPR-repressed genes 17 showed significantly increased levels, 6 unchanged and 27 significantly reduced expression levels during biotrophic development [22] (S3 Table). The large overlap of the UPR- and plant-induced genes provides further evidence for the central role of the UPR during biotrophic development of *U*. *maydis*.

### Clp1 modulates UPR core gene expression

**Fig 2:**
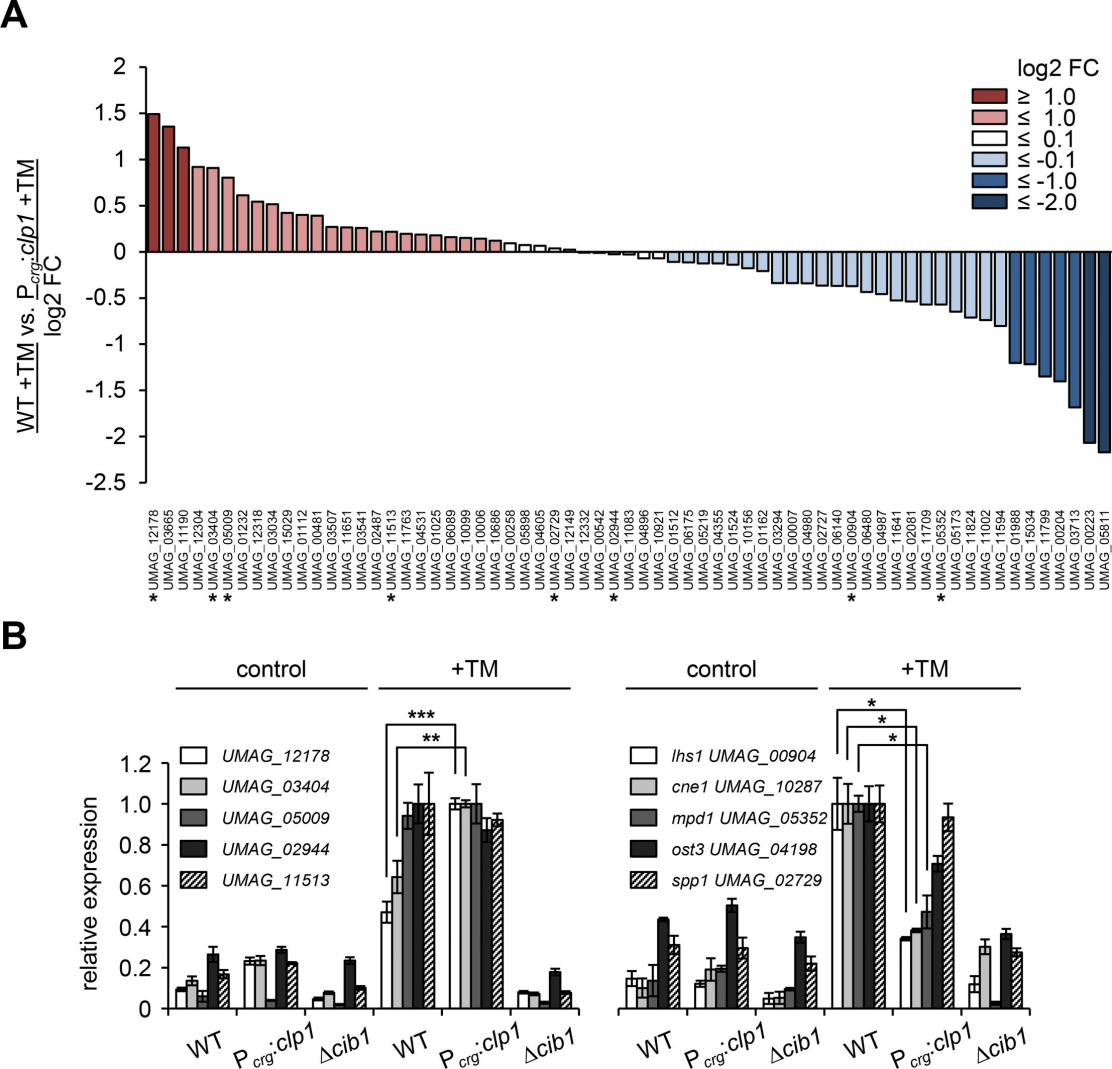
Overview of Clp1-mediated modulation of UPR core genes. **(A)** Overview of log2 transformed fold changes comparing UPR core gene expression after TM treatment in the *clp1* expressing P_*crg*_:*clp1* strain vs. WT. Red color indicates increased expression, blue color indicates reduced expression in the P_*crg*_:*clp1* strain, respectively (log2FC>1). Light colors indicate tendencies for higher/lower expression. Asterisks (*) indicate genes that were further analyzed in (B). **(B)** qRT-PCR analysis of UPR core genes in strains JB1 (WT) and derivatives harboring *clp1* under the control of the arabinose inducible *crg1* promoter (P_*crg*_:*clp1*) or in the *cib1* deletion strain (Δ*cib1*). Strains were cultured in YNBG to an OD_600_ of 0.25 and shifted to YNBA medium to induce *clp1* expression in strains containing P_*crg*_:*clp1*. TM was added to a final concentration of 5 μg/mL, and strains were further incubated for 4 hours. The Δ*cib1* strain served as negative control to test for unspecific effects of TM treatment on gene expression. Expression is shown relative to highest expression value. Expression values represent the mean of three biological replicates with two technical duplicates each. *eIF2b* (*UMAG_04869*) was used for normalization. Error bars represent the SD. Statistical significance was calculated using Student’s *t* test. *P value ≤ 0.05, **P value ≤ 0.01 and ***P value ≤ 0.001.

To address the effects of Clp1 on the UPR we compared UPR core gene expression between WT (JB1) and P_*crg*_:*clp1* (UVO151) strains after TM-mediated UPR activation. We reasoned that monitoring gene expression changes of the UPR core gene set might be most informative as their regulation is unlikely to involve additional factors. Expression of only 10 of the 65 UPR core genes was affected more than 2-fold (log2FC ≥+/-1) (7 repressed, 3 induced) by *clp1*. In addition, expression of 32.3% (21 of 65) of the UPR core genes trended to be higher in UVO151 (log2FC = 0.92 to 0.1), whereas expression of 35.4% (23 of 65) of the genes trended to be decreased (log2FC = -0.1 to -0.8) and expression of 17% (11/65) remained completely unaffected (log2FC = -0.1 to 0.1) (Fig 2A and S1 Table). Although these effects appear marginal, comparison of normalized read counts per kilobase of transcript, per Million of mapped reads (RPKM) of all 65 UPR core genes revealed a drop of more than one fourth (26%) in UVO151 (23919) compared to JB1 (32380). Overall differences in UPR gene expression were modest between both strains. We thus analyzed by qRT-PCR expression of 8 UPR core genes (increased by *clp1*: *UMAG_12178*, *UMAG_03404*; not affected: *UMAG_05009*; *UMAG_02944*, *UMAG_11513*, *UMAG_02729-spp1*; decreased by *clp1*: *UMAG_00904-lhs1*, *UMAG_5352-mpd1*) and 2 additional UPR marker genes (*UMAG_10287-cne1* and *UMAG_05352-ost3*) using cDNA that was generated in independent experiments (n = 3). Expression of all genes was significantly increased by TM treatment (p<0.05) in strain JB1 and remained at basal levels under identical conditions in the Δ*cib1* background (Fig 2B). All genes showed a similar modulation of gene expression by Clp1 in qRT-PCR and RNAseq analysis, confirming our initial results.

### Clp1 does not affect DNA-binding specificity of Cib1

Protein-protein interactions between accessory proteins and bZIP transcription factors can influence gene expression in many different ways [50], including modulation of the protein-DNA interaction. To identify direct Cib1 targets and to test if the interaction with Clp1 affects DNA binding by Cib1, we performed chromatin immunoprecipitation followed by massively parallel DNA sequencing (ChIP-seq) analysis. We expressed a functional Cib1-3xHA fusion protein [46] under the control of the endogenous promoter from the endogenous genomic locus in the WT (JB1) and P_*crg*_:*clp1* strain (UVO151). Four hours after TM-mediated UPR induction chromatin was isolated, followed by immunoprecipitation (ChIP) using anti-HA beads and sequencing of enriched DNA. The experiment was performed with two biological replicates for each strain (n = 2). In addition, input DNA and DNA unspecifically bound to HA-agarose beads (mock) was sequenced to identify and correct for binding or sequencing bias. Peak calling was performed using peakZilla [51], providing a peak-score (Δ normalized reads (IP-input) x distribution score) conflating true positive probability of DNA binding and estimation of DNA binding strength. Scores of individual peaks were accumulated when identified on a single promoter (1.5 kb upstream of translation start site (tss)) and at least one peak score was ≥40, to yield promoter scores. Promoters were filtered with a promoter score cut-off of ≥100. 217 promoters were bound by Cib1-3xHA in both strains, and 63 and 188 promoters were only bound in the WT (JB1*cib1-3xHA*) or P_*crg*_:*clp1* (UVO151*cib1-3xHA*) strain, respectively. 41 and 46 promoters of UPR core genes had scores ≥100, in WT and P_*crg*_:*clp1*, respectively. Importantly, within the top twenty list of promoters with the highest scores we identified in both strains the known UPR genes *bip1*, *cib1*, *pdi1*, *ero1*, *lhs1*, *pmr1*, *lhs1* and *dnj1* (S4 Table). We further tested binding of Cib1 to the promoters of *cib1* and *ero1* by ChIP-qPCR analysis (S2 Fig). In comparison to the negative control *eIF2b*, amplicons corresponding to the respective promoter regions were highly enriched, confirming binding to both promoters and the previously postulated autoregulation of *cib1* gene expression by the Cib1 protein [36].

**Fig 3:**
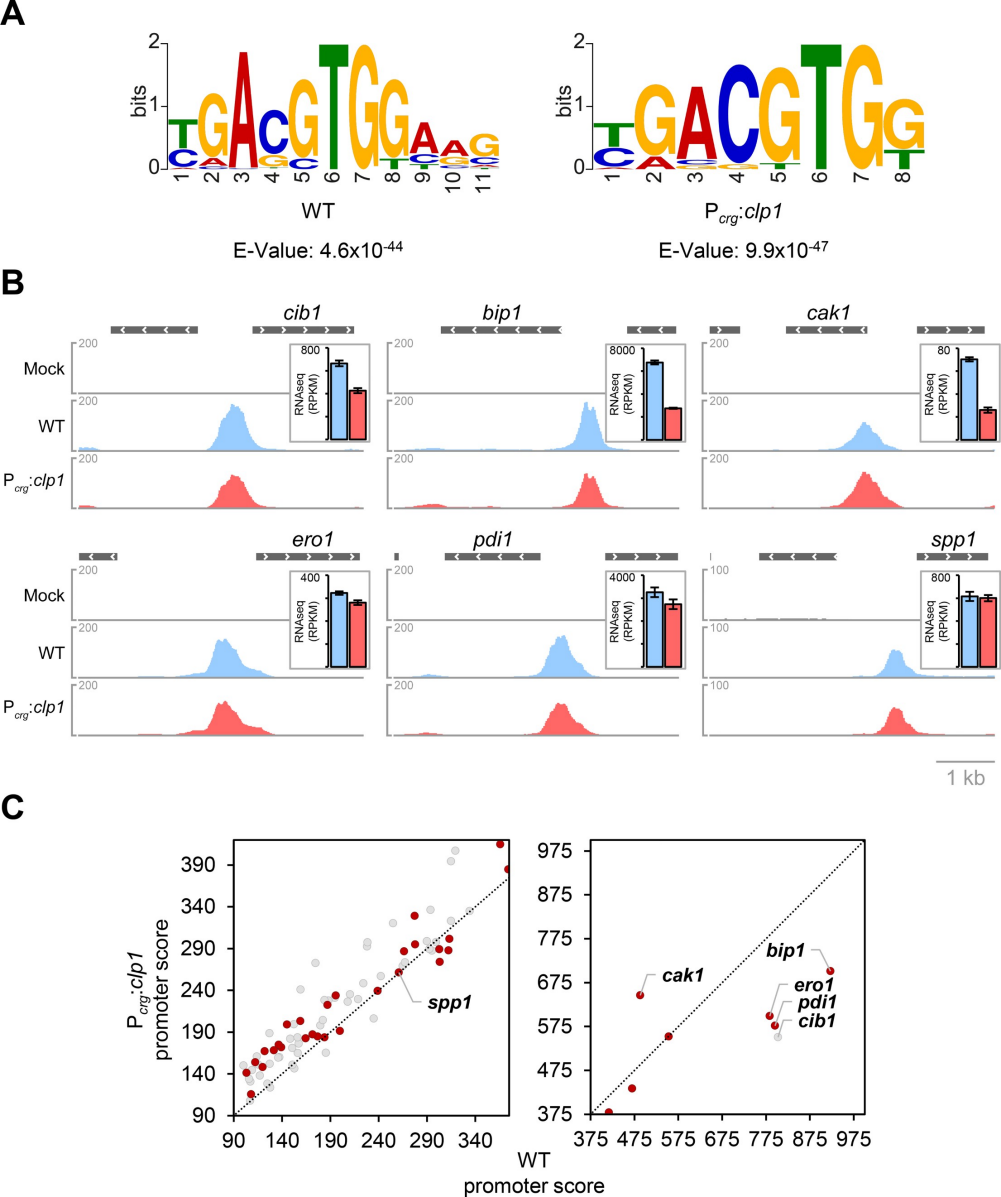
ChIPseq analysis after TM-mediated UPR induction. **(A)** Sequence motifs enriched in JB1*cib1-3xHA* (WT) and UVO151*cib1-3xHA* (P_*crg*_:*clp1*) strains using MEME (Multiple EM for Motif Elicitation)-ChIP. *U*. *maydis* strains JB1 (Mock), JB1*cib1-3xHA* (WT) and UVO151*cib1-3xHA* (P_*crg*_:*clp1*) were grown in CM supplemented with 1% glucose (CMG) to an OD_600_ of 0.25 and shifted to CM supplemented with 1% arabinose (CMA), to induce expression of *clp1* in strain UVO151*cib1-3xHA* (P_*crg*_:*clp1*). TM was added to a final concentration of 5 μg/ml for UPR induction and strains were further incubated for 4 hours at 28°C. Fasta files were generated from peaks in the promoters of the 91 UPR core genes identified as direct Cib1 targets in both strain backgrounds and subjected to MEME-ChIP analysis. Probability of nucleotide-occurrence in the consensus motif is reflected by height of the letters compared to the entire motif. Stacked letters indicate that more than one nucleotide can occur at the position in the motif. Calculation of E-values is based on the log likelihood ratio, width, sites, the background letter frequencies, and the size of the training set. **(B)** Visualization of Cib1 binding to promoters of UPR genes obtained by ChIPseq analysis. ChIPseq data was visualized using normalized BigWig files data and the Integrative Genomics Viewer (IGV). Peak profiles represent normalized read counts derived from two biological replicates in JB1*cib1-3xHA* (blue) and UVO151-*cib1-3xHA* (red) in comparison to the untagged mock control (grey). Insets depict gene expression as determined by RNAseq analysis of respective genes in reads per kilobase of transcript, per million mapped reads (RPKM) as mean of three biological replicates. Error bars depict the SD. The scale bar corresponds to 1 kb. **(C)** Scatter plot of promoter scores of UPR core genes derived from ChIPseq analysis in JB1*cib1-3xHA* (WT, x-axis) and UVO151*cib1-3xHA* (P_*crg*_:*clp1*, y-axis). Individual peak scores were calculated by peakZilla (https://github.com/steinmann/peakzilla) and accumulated to a promoter score if more than one peak was identified on a single promoter (1.5 kb upstream of translation start site (tss)). Red dots indicate UPR core genes (log2FC≥2), whereas grey dots indicate UPR regulated genes filtered under less stringent criteria (log2FC≥1).

MEME-ChIP analysis of peaks derived from promoters specifically bound by Cib1-3xHA in WT or the P_*crg*_:*clp1* strain did not reveal a clear binding motif. By contrast, when peaks were derived from UPR core gene promoters that were bound in both, WT and P_*crg*_:*clp1* strains (log2FC≥1, n = 114 peaks, n = 91 promoters), an overrepresented CRE3-like binding motif T/CGACGTGGAAG (E = 4.6e^-44^) was identified in WT background (Fig 3A) [52]. This motif is highly similar to the unfolded protein response element (UPRE) bound by Xbp1 in human (GATGACGTGGC, E = 2.0e^-8^) [52, 53] and almost perfectly matches the Cib1-binding site consensus sequence (reverse complement: CGACGTGGCA) in the promoter regions of *pit1/2* and *tin1-1* [46]. However, by ChIPseq only binding to the *tin1-1* promoter was detectable (S3 Fig). When MEME-ChIP analysis was performed on peaks derived from the *clp1* expressing P_*crg*_:*clp1* strain a largely overlapping TGACGTGG motif was highly enriched (E = 9,9e^-47^), lacking only the terminal AAG triplet of the motif identified in the WT strain (Fig 3A). Consistently, visual inspection of ChIPseq data using the integrated genomics viewer tool (IGV) revealed that shapes and locations of peaks were conserved between strains as illustrated by the representative examples (Fig 3B).

Of the 65 UPR core gene (log2FC = 2) promoters 41 were bound by Cib1-3xHA in WT and 46 were bound in the P_*crg*_:*clp1* strain, with an overlap of 37 promoters (S4 Table). Comparison of promoter scores revealed that promoters with the four highest scores in WT (*bip1*, *cib1*, *pdi1*, *ero1*) showed the strongest score decrease in the P_*crg*_:*clp1* strain in conjunction with reduced expression levels in UVO151 (P_*crg*_:*clp1*) in comparison to the JB1 (WT) control (Fig 3C and S1 Table). Promoter scores and expression levels of *spp1*, predicted to encode a signal peptide peptidase, correlated as well when compared between both strains. In contrast, for *UMAG_11799* (*cak1*) these were inversely correlated (Fig 3B). In summary, our data suggest that modulation of UPR core gene expression is not related to an altered DNA binding specificity of Cib1 and that modulation of UPR gene expression by Clp1 might involve other mechanisms.

### Clp1 affects abundance, stability and phosphorylation of Cib1

**Fig 4:**
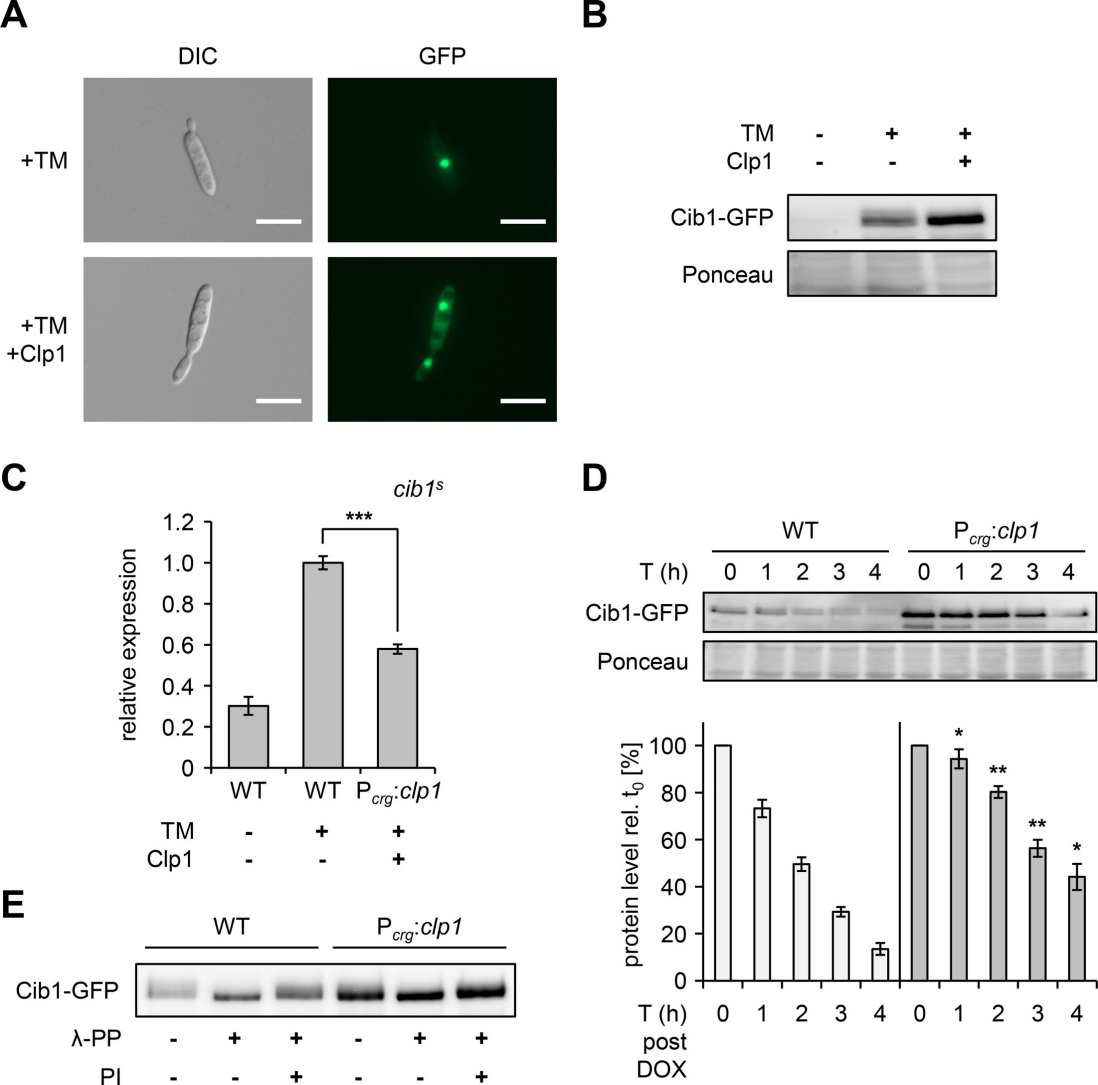
Clp1 alters stability and phosphorylation of Cib1. **(A)** Cib1-GFP was monitored by fluorescence microscopy. Cells of indicated strains were incubated as described in Fig 4B, as indicated. A nuclear localized GFP signal was observed in cells treated with TM, indicating expression of the Cib1-GFP fusion protein. An additional cytoplasmic fluorescence signal was observed in the UVO151 background, expressing *clp1*. Exposure time was set to 500 ms. Morphology of *U*. *maydis* cells was visualized by differential interference contrast (DIC) microscopy. Scale bar = 10 μm. **(B)** Analysis of Cib1-GFP expression by Western hybridization in response to TM treatment in *U*. *maydis* strains JB1*cib1-GFP* (WT) and the *clp1*-expressing derivative UVO151*cib1-GFP* (P_*crg*_:*clp1*). Protein extracts were prepared from cells incubated in liquid CMA to induce *clp1* expression in the P_*crg*_:*clp1* strain background. Where indicated, TM was added to a final concentration of 5 μg/ml and strains were further incubated for 4 hours at 28°C. Cib1-GFP was detected by a GFP-specific antibody. Ponceau S-stained bands served as loading control. **(C)** qRT-PCR analysis of the spliced *cib1*^*s*^ transcript in response to Clp1 induction. RNA was prepared from exponentially growing *U*. *maydis* JB1 (WT) and UVO151 (P_*crg*_:*clp1*) strains cultured in YNBA liquid medium supplemented with 5 μg/mL TM for 4 hours. Primers specifically detecting the spliced *cib1* transcript (*cib1*^*s*^) were used. *eIF2b* was used for normalization. Expression values represent the mean of three biological replicates with two technical duplicates each. Error bars represent the SD. Statistical significance was calculated using Student’s *t* test. ***P value ≤ 0.001. **(D)** Analysis of Cib1-GFP protein stability by promoter shut-off using the Tet-off system. Strains JB1*cib1-GFP* (WT) and UVO151*cib1-GFP* (P_*crg*_:*clp1*) were grown to an OD of 0.35 in CMG liquid medium and shifted to CMA containing TM (5 μg/ml) to induce expression of *clp1* and Cib1-GFP, respectively. After 4 hours, doxycycline was added (f.c. 10 μg/ml) to block transcription of *cib1-GFP*. Protein extracts were prepared from samples taken directly before the addition of doxycycline (T0) and 1 h (T1), 2 h (T2), 3 h (T3) and 4 h (T4) after doxycycline treatment. Cib1-GFP was detected by Western hybridization. Ponceau S staining of the membrane was used as loading control and for normalization of Cib1-GFP levels. Protein levels at T1, T2, T3 and T4 were determined relative to T0 using ImageJ. Values represent the mean of 3 biological replicates and error bars indicate SD. Statistical significance was calculated using Student’s *t* test. *P value ≤ 0.05 and **P value ≤ 0.01. **(E)** Western hybridization after phosphatase treatment using lambda phosphatase (λ-PP). Protein extracts were prepared from exponentially growing cells as described for Fig 4A. Cib1-GFP was immunoprecipitated using GFP-trap MA beads (Chromotek) and phosphatase treatment was performed on beads with lambda phosphatase (λ-PP). Phosphatase inhibitor (PI) was used to inhibit λ-PP and Cib1-GFP was detected using a GFP-specific antibody.

To further explore how Clp1 affects UPR gene expression we first analyzed the effect of Clp1 on Cib1 protein localization. We visualized the subcellular localization of a functional Cib1-GFP fusion protein expressed under the control of its endogenous promoter from the endogenous genomic locus in strains JB1*cib1-GFP* (WT) and UVO151*cib1-GFP* (P_*crg*_:*clp1*), by fluorescence microscopy. Four hours after TM treatment Cib1-GFP was detectable in the nucleus of WT cells and was in addition localized to the cytoplasm when *clp1* was expressed (P_*crg*_:*clp1*) (Fig 4A). Next, we tested by Western hybridization whether Cib1-GFP levels were altered by *clp1* expression. Cib1-GFP was detectable after TM treatment and higher levels of Cib1-GFP were detected in the *clp1* expressing strain (P_*crg*_:*clp1*) in comparison to the WT control (Fig 4B). To test if increased abundance of the fusion protein correlated with increased expression or splicing of *cib1* mRNA we used qRT-PCR analysis to determine transcript levels of the spliced *cib1*^*s*^ mRNA. This revealed a TM-dependent increase of *cib1*^*s*^ levels in both strains. However, contrary to the Cib1 protein levels, transcript abundance was significantly lower in the *clp1*-expressing strain (P_*crg*_:*clp1*) when compared to the WT control (Fig 4C). We therefore concluded that Clp1 affects abundance of Cib1 posttranscriptionally.

To test if the increased abundance of Cib1-GFP is related to altered protein stability, we performed promoter shut-off assays using the doxycycline-repressible Tet-off system [54]. To this end, we expressed Cib1-GFP under the control of the Tet-promoter from the native genomic locus. We induced ER stress with TM for 4 hours, blocked transcription of *cib1-GFP* with doxycycline (10 μg/ml) and monitored Cib1-GFP levels over time. We quantified protein levels relative to T0 and observed that Cib1-GFP levels decreased significantly slower in the *clp1* expressing strain when compared to the WT control (Fig 4D). This observation was as well confirmed by cycloheximide chase assays (S1 Fig). This indicates that Cib1-GFP is stabilized by Clp1 and that this accounts for the increased abundance of Cib1-GFP.

In addition to the differences in Cib1-GFP levels we noticed that the fusion protein displayed mobility shifts in Western hybridization experiments that might be caused by posttranslational modifications (Fig 4B). Protein phosphorylation often precedes ubiquitin-dependent protein degradation [55]. Since mobility shifts were more pronounced in the WT when compared to the *clp1* expressing strain (P_*crg*_:*clp1*) we speculated that stability of Cib1-GFP might be related to phosphorylation of the fusion protein. We tested this assumption by phosphatase treatment of protein extracts prepared from WT and *clp1* expressing cells. Phosphatase treatment prevented the mobility shift in extracts derived from WT (JB1) cells, and this could be blocked by addition of phosphatase inhibitor. By contrast, only minor effects were observed when protein extracts were derived from the *clp1* expressing (P_*crg*_:*clp1)* strain (Fig 4E). These results suggest that Cib1-GFP is phosphorylated and that phosphorylation is reduced by Clp1. In summary, our data demonstrate that Clp1 influences phosphorylation, stability and localization of Cib1-GFP. We assume that these processes are interdependent and connected to the Clp1-mediated modulation of UPR gene expression.

### Gene deletion screening of UPR core genes identifies Spp1 as novel virulence factor in *U*. *maydis*

Modulation of the UPR by Clp1 enables long-term activity during biotrophic growth of *U*. *maydis* [36]. Cib1 and Clp1 are specifically expressed after plant penetration and throughout all subsequent stages of biotrophic growth. Hence, it is conceivable that the UPR is constitutively modulated *in planta* and that the modulation of UPR core gene expression represents a promising read-out to identify potential virulence or ER stress related factors.

We selected 32 candidate genes on basis of an increased or stable expression in *clp1* expressing strains when compared to the WT (Fig 2A, genes marked in red and white) and screened potential candidates for altered virulence and ER stress resistance. To this end, we deleted the open reading frame (ORF) of a total of 29 UPR core genes in the solopathogenic SG200 strain background [56]. We were not successful in our attempt to generate deletion strains for three additional genes (*UMAG_00481*, *UMAG_06089*, and *UMAG_15029*), all of which are predicted to encode signal peptidase components, suggesting that these might be essential for growth. Surprisingly, none of the 29 deletion strains showed reduced ER stress resistance (S4 Fig) and 26 of the 29 deletion strains displayed no alteration of virulence (S5 Fig). Single deletions of the two genes *UMAG_12178*, encoding a protein related to 5-carboxyvanillate-decarboxylases and *UMAG_11083* encoding a p24-like protein, resulted in slightly reduced virulence. Strikingly, only deletion of *UMAG_02729*, predicted to encode a signal peptide peptidase resulted in the loss of virulence and complete absence of tumor formation (S5 Fig). This suggests that UMAG_02729 (hereafter referred to as Spp1) represents a major virulence factor that is connected to the UPR in *U*. *maydis*.

### Spp1 is specifically required for biotrophic development of *U*. *maydis*

The Spp1 protein is predicted to function as signal peptide peptidase (SPP). SPPs are ER-membrane localized aspartyl-proteases that are found in all eukaryotes including fungi, protozoa, plants and animals and catalyze intramembrane proteolysis at the ER membrane. SPP substrates include signal peptide remnants (cleavage products of signal peptidases), ER membrane-bound transcription factors or high-affinity transporters [28, 29, 33, 57, 58]. *spp1* belongs to the UPR core genes identified under stringent filtering criteria (7.9-fold increased by TM) (Fig 1B and S1 Table) and expression of *spp1* is as well strongly induced *in planta* (11-fold at 2 dpi vs. axenic) (Fig 1D) [22, 36]. *spp1* levels were not affected by Clp1-mediated UPR modulation (Fig 2A and 2B) and promoter scores derived from ChIPseq of WT and *clp1* expressing strains were almost identical (promoter score: 261) (Fig 3B and 3C and S4 Table). Hence, we discovered a direct regulation of *spp1* expression by the UPR, which to the best of our knowledge, has not been described in other organisms.

**Fig 5:**
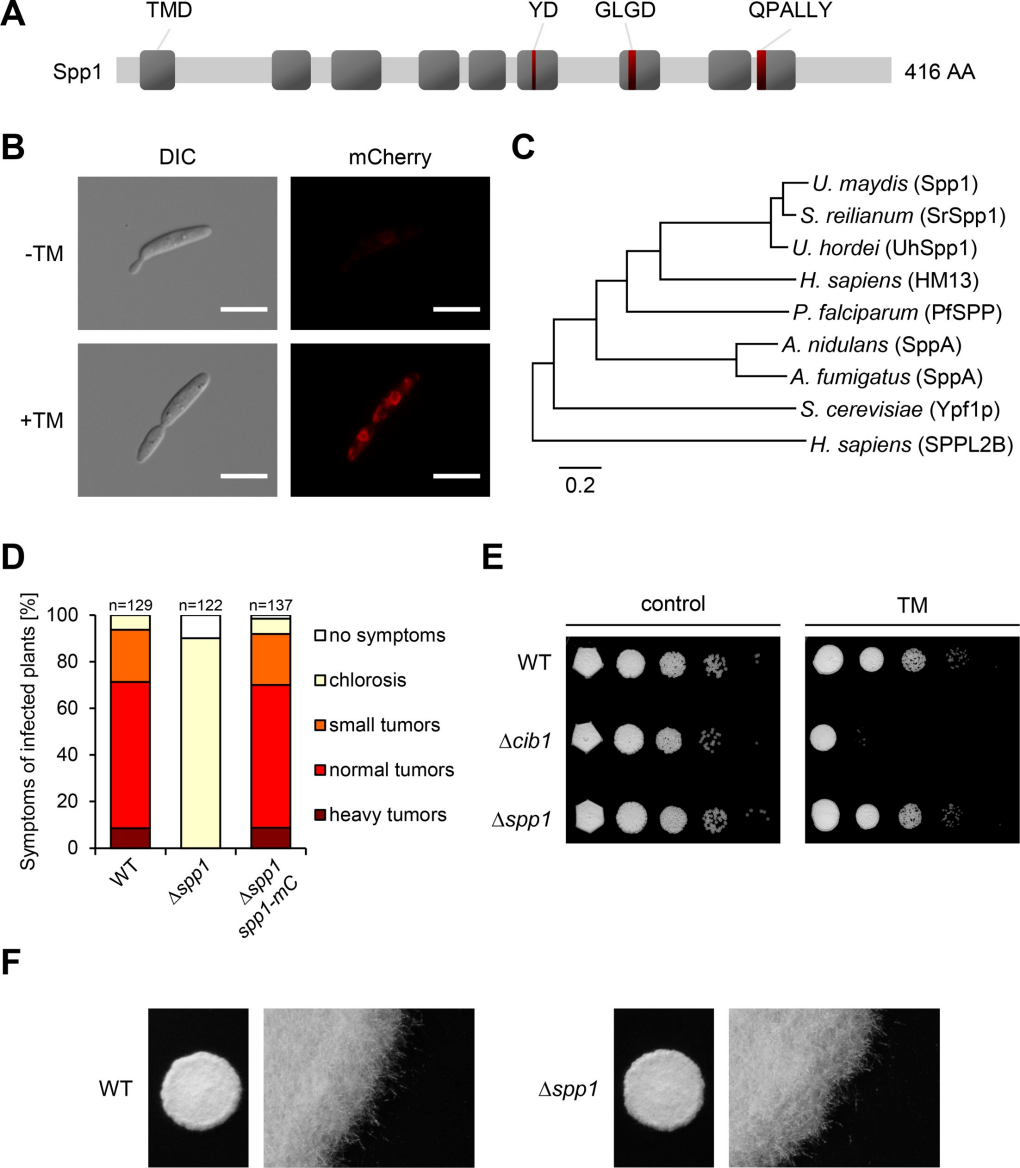
Spp1 is a functionally conserved signal peptide peptidase and required for pathogenicity. **(A)** Schematic representation of the Spp1 domain structure. Grey boxes represent transmembrane domains (TMD). Red bars within TMDs mark the conserved YD, GLGD and QPALLY motifs. **(B)** Expression of the Spp1-mCherry fusion protein was monitored by fluorescence microscopy 2 h after TM-mediated UPR induction in strain SG200Δ*spp1*-*P*_*spp1*_:*spp1-mCherry*. Exposure time was set to 500 ms. Cellular morphology was visualized by DIC microscopy. Scale bars = 10 μm. **(C)** Phylogenetic tree of *U*. *maydis* Spp1 and predicted orthologs from *Sporisorium reilianum* (SrSpp1), *Ustilago hordei* (UhSpp1), *Homo sapiens* (HM13), *Plasmodium falciparum* (PfSPP), *Aspergillus nidulans* (SppA), *Aspergillus fumigatus* (SppA) and *Saccharomyces cerevisiae* (Ypf1p). *H*. *sapiens* SPPL2B (Signal peptide peptidase-like 2B) was used as an outgroup. Construction of the phylogenetic tree was performed using the MEGA X software (https://www.megasoftware.net) by the Maximum Likelihood method based on sequence alignment by the MUSCLE algorithm. **(D)** The haploid pathogenic strain SG200 (WT) and derivatives were inoculated into 7 day-old maize seedlings. Disease symptoms were rated 8 days after inoculation (dpi) and grouped into categories depicted on the right. n represents the total number of inoculated plants in three independent experiments. **(E)** ER stress assay of strain SG200 (WT) and derivatives to analyze ER stress resistance. The Δ*cib1* strain was used as negative control. 10-fold serial dilutions of indicated strains were spotted on YNBG solid medium. TM was added to a final concentration of 0.5 μg/ml. Plates were incubated for 48 h at 28° C. **(F)** *U*. *maydis* strain SG200 (WT) and the Δ*spp1* derivative were spotted on charcoal containing (1% w/v) potato-dextrose (PD) plates to induce filament formation. Plates were incubated for 24 h at 28°C and photographed. White fuzzy filaments indicate formation of *b*-dependent filaments.

Spp1 displays the characteristic SPP domain structure with 9 predicted trans membrane (TM) domains (http://www.cbs.dtu.dk/services/TMHMM/) [59], harboring the highly conserved YD and GLGD motifs important for catalytic activity and the QPALLY motif putatively conferring substrate specificity [29] (Fig 5A and S6 Fig). To visualize the subcellular localization of Spp1 we expressed the protein as a C-terminal mCherry (mC) fusion under the control of the native *spp*1 promoter in the SG200Δ*spp1* background. In accordance with the predicted function, fluorescence microscopy revealed that 2 hours after TM-mediated UPR induction Spp1-mC localized to structures resembling the perinuclear and cortical ER (Fig 5B). By contrast, no accumulation of Spp1-mC fusion protein was observed in the Δ*cib1* background under these conditions (S7 Fig), corroborating that the accumulation of Spp1-mC requires *cib1*-dependent UPR induction and is not a side effect of TM treatment. Expression of *spp1-mC* under the control of the constitutive active *otef* promoter revealed that localization of Spp1-mC is similar in WT and Δ*cib1* strain background (S7 Fig). Phylogenetic analysis revealed that outside of the *Ustilaginales* Spp1 is related to human HM13 (identity: 35%, similarity: 52%, E-value: 2e^-72^) and *Plasmodium falciparum* PfSPP (identity: 33%, similarity: 49%, E-value: 6e^-55^) and more distantly related to SppA from *Aspergillus* species (identity: 28%, similarity: 43%, E-value: 9e^-50^) and yeast Ypf1p (identity: 32%, similarity: 52%, E-value: 2e^-32^) (Fig 5C).

To retest our screening results we performed plant infection assays with WT (SG200), Δ*spp1* and the Δ*spp1-spp1-mC* complementation strain, in which the *spp1-mCherry* fusion construct was integrated into the *ip*-locus of strain SG200Δ*spp1* and expressed under the control of the native *spp1* promoter. The loss of virulence in the Δ*spp1* mutant was fully complemented by expression of Spp1-mCherry (Spp1-mC), indicating that the fusion protein is functional (Fig 5D). Analysis of vegetative growth under axenic conditions, ER stress resistance, cell wall stress resistance or the ability to form *b*-dependent filaments on charcoal containing solid medium did not reveal differences between WT (SG200) and the Δ*spp1* (SG200Δ*spp1*) derivative (Fig 5E and 5F and S8 Fig).

**Fig 6:**
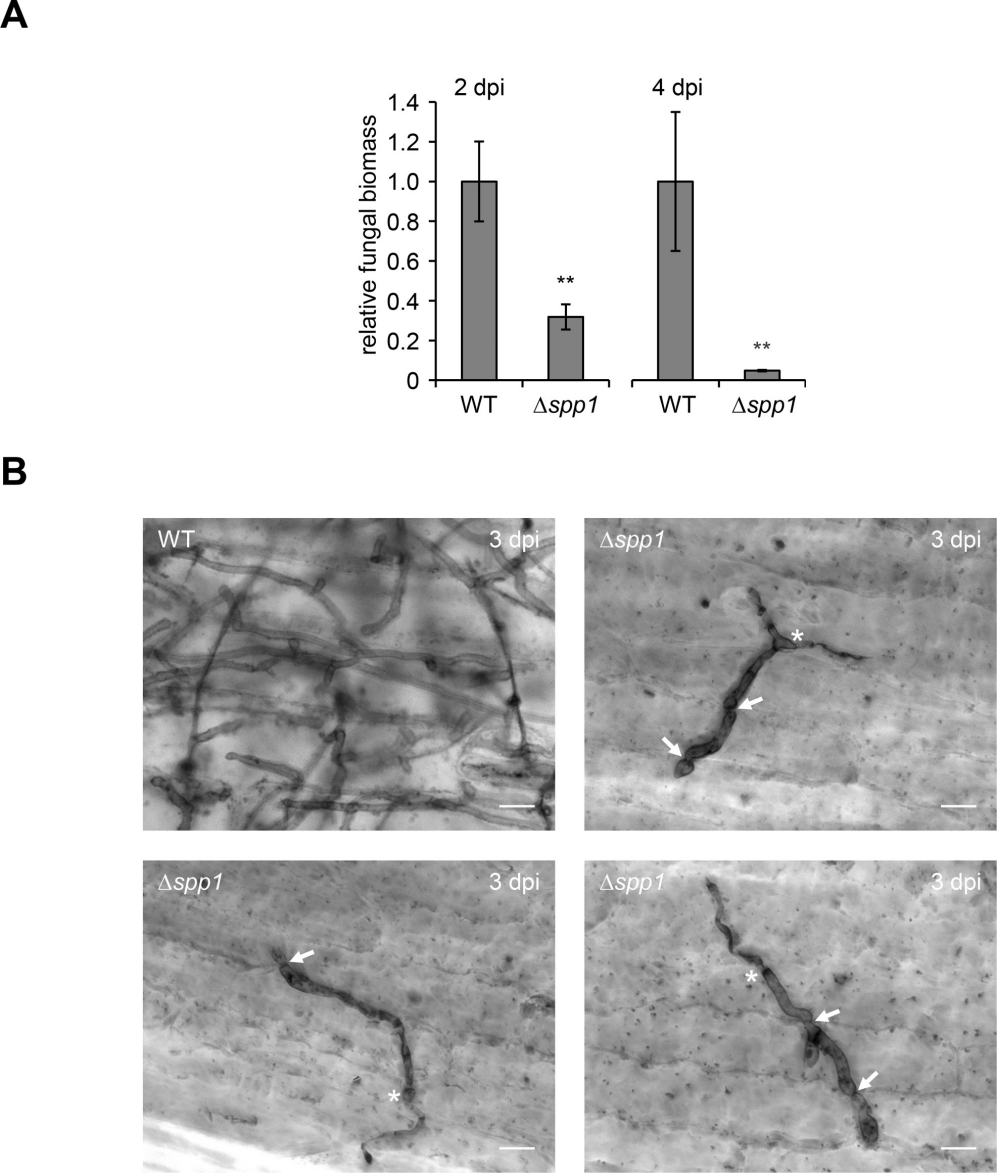
Spp1 is important for proliferation *in planta*. **(A)** Quantification of fungal biomass by quantitative PCR analysis. Genomic DNA was extracted from maize seedlings inoculated with indicated strains at 2 and 4 dpi. Relative fungal biomass was calculated using the *U*. *maydis* specific *mfa1* gene as fungal marker and the *Zea mays* specific glyceraldehyde 3-phosphate dehydrogenase (*GAPDH*) as plant marker. Values reflect the ratio of fungal/plant DNA relative to plants infected with the WT. Values represent the mean of three biological replicates with two technical duplicates each. Error bars represent the SD. Statistical significance was calculated using Student’s *t* test. **P value ≤ 0.01. **(B)** Fungal proliferation of SG200 (WT) and the Δ*spp1* was investigated by Chlorazol Black E staining of infected leaf samples at 3 dpi. WT strains showed extensive proliferation *in planta*, whereas the Δ*spp1* mutant showed strongly reduced proliferation after plant penetration. Asterisks mark the site of plant penetration and arrows indicate the points of plant cell traversal by fungal hyphae. Scale bar = 10 μm.

Since formation of infectious filaments was not affected in Δ*spp1* strains we investigated at which stage of pathogenic development Δ*spp1* mutants are blocked. To this end, we quantified the fungal biomass at 2 and 4 dpi, using the *mfa1* gene as fungal marker, in maize plants inoculated with either the WT (SG200) or the Δ*spp1* deletion mutant. This revealed that at both time points significantly less fungal biomass was produced in plants inoculated with the Δ*spp1* mutant in comparison to the WT (SG200) control (Fig 6A). To assess the fungal morphology during biotrophic development, we stained infected leaf tissue 3 dpi with Chlorazol Black E and analyzed the samples by microscopy (Fig 6B). While the WT (SG200) strain showed extensive proliferation, hyphal branching and clamp cell formation, deletion mutants of *spp1* were severely impaired. Δ*spp1* strains displayed filamentous growth and formed appressoria that penetrated the leaf surface. However, proliferation of fungal hyphae after plant penetration was highly reduced and in most cases restricted to the epidermal cell layer. In addition, hyphae of the Δ*spp1* strain showed altered intercellular growth with bulbous enlargements formed prior and after plant cell wall traversal and constrictions at the point of plant cell wall passage (Fig 6B, white arrows). Together, this demonstrates that Spp1 is required for proliferation *in planta*, which might be connected to defects in intercellular growth.

### Spp1 is an evolutionary conserved SPP that requires its catalytic activity to cause disease

**Fig 7:**
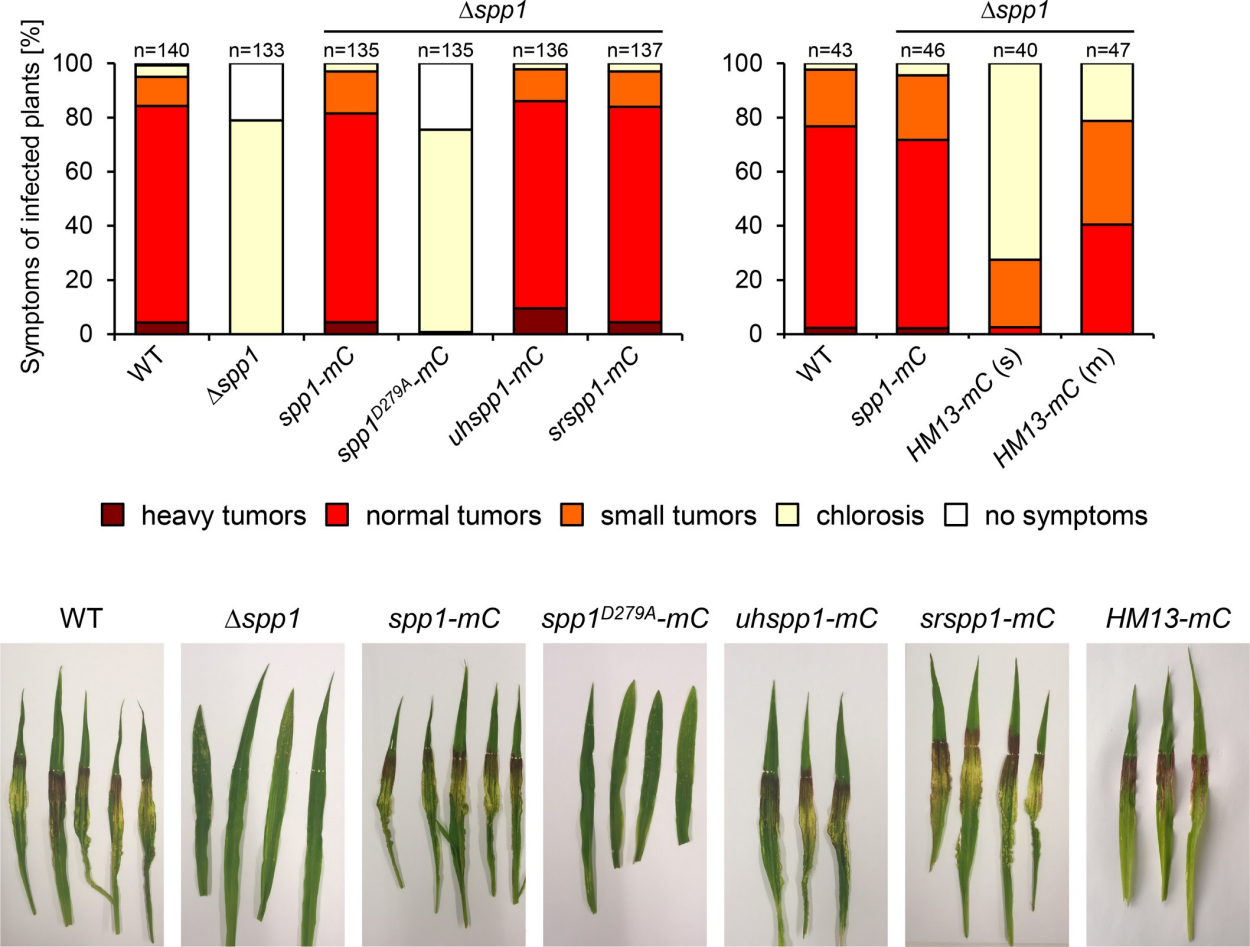
Expression of fungal and human Spp1 orthologs restores pathogenicity of Δ*spp1* mutants. *U*. *maydis* strains SG200 (WT), SG200Δ*spp1* (Δ*spp1*) and derivatives were inoculated into 7-d-old maize seedlings. In Δ*spp1* derivatives, predicted homologs or the catalytically inactive Spp1^D279A^ mutant protein were expressed as mCherry (mC) fusion under the control of the constitutively active *otef* promoter. Disease symptoms were scored 8 dpi. n represents the total number of inoculated plants derived from three (left) or two (right) independent experiments. For complementation tests using *H*. *sapiens HM13*, Δ*spp1* strains harboring single (s) and multiple (m) integrations of the *HM13-mCherry* (*HM13-mC*) fusion construct were used. Leaf samples for macroscopic analysis were photographed at 8 dpi.

To test for functional conservation between Spp1 and potential orthologs in *Sporisorium reilianum*, *Ustilago hordei*, *Aspergillus nidulans*, *S*. *cerevisiae* and *Homo sapiens*, respective genes were expressed as C-terminal mCherry (mC) fusion under the control of the constitutive *otef* promoter in *U*. *maydis* strain SG200Δ*spp1* (Δ*spp1*). In addition, we tested if catalytic activity of Spp1 is required for virulence of *U*. *maydis* by expressing the Spp1^D279A^ mutant, harboring a D>A exchange in the highly conserved GLGD motif that is known to abolish catalytic activity [29], in the Δ*spp1* strain. Expression of the fusion proteins was tested by Western hybridization using anti-mCherry antibodies. Expression of all fusion proteins was detectable with the exception of *S*. *cerevisiae* Ypf1p-mC and *Aspergillus nidulans* SppA-mC (S9 Fig). Previous studies revealed premature polyadenylation of transcripts in *U*. *maydis* if GC content or codon usage was explicitly different from *U*. *maydis* [54]. It appears likely that the failure to express Ypf1p-mC and SppA-mC might be prevented by this mechanism. Expression of Spp1 orthologs from *S*. *reilianum* and *U*. *hordei* fully rescued the virulence defect of the Δ*spp1* mutant, whereas expression of *H*. *sapiens* HM13-mC suppressed the virulence defect of the Δ*spp1* mutant only partially, but in a dose-dependent manner (Fig 7). Importantly, expression of Spp1^D279A^ did not rescue the virulence defect of the Δ*spp1* mutant, as infected plants were indistinguishable from those infected by the Δ*spp1* progenitor strain (Fig 7). In conclusion, our data strongly suggest that Spp1 is a bona fide SPP, homologous to HM13 and that cleavage of SPP substrates is essential for virulence of *U*. *maydis*.

### Central ERAD components, the putative sterol regulatory element binding protein (SREBP) Srb1 and the *U*. *maydis* heme oxygenase are dispensable for virulence

**Fig 8:**
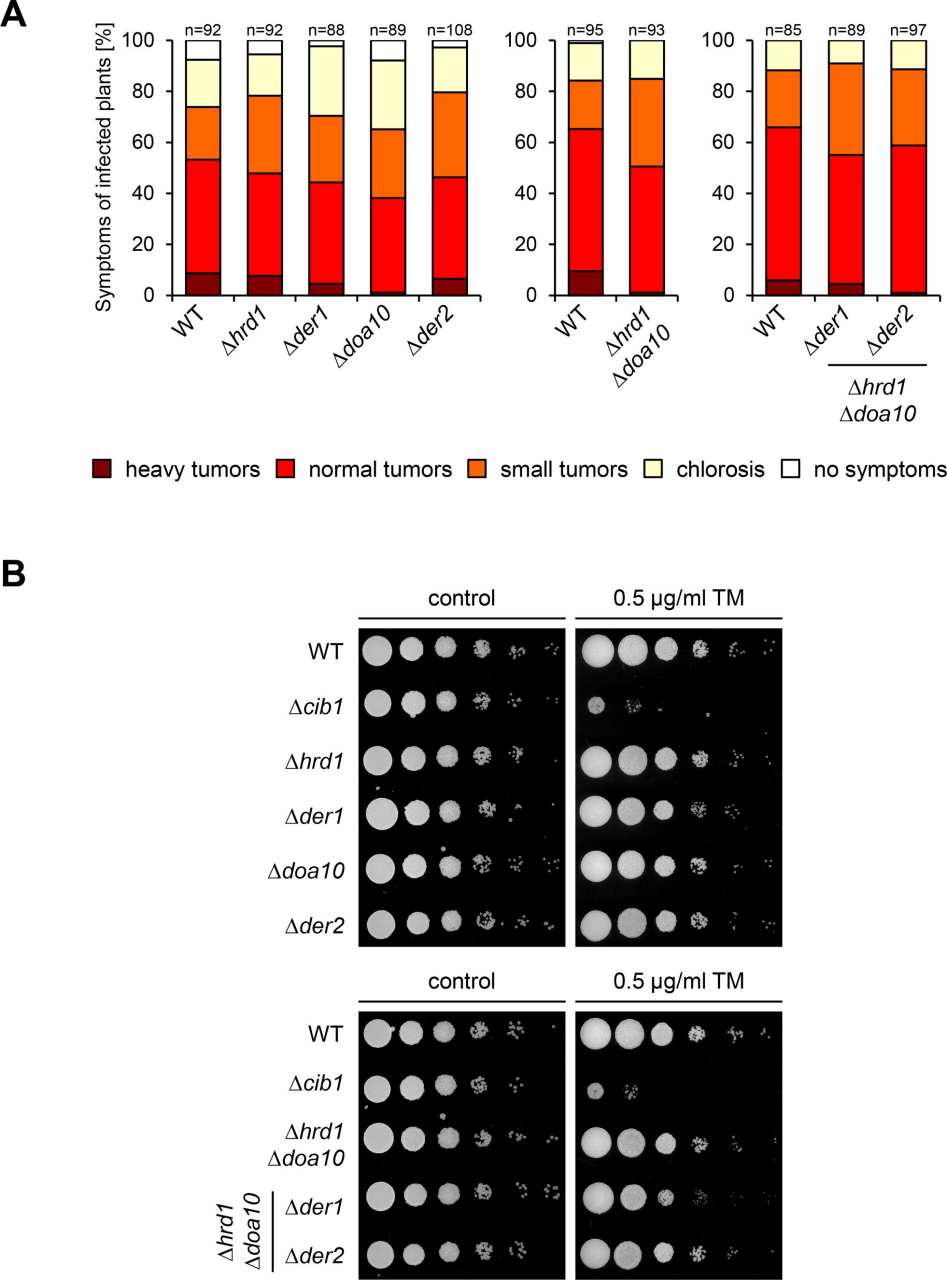
Deletion of conserved ERAD components does not affect ER stress resistance or virulence of *U*. *maydis*. **(A)** *U*. *maydis* strain SG200 (WT) and derivatives were inoculated into 7 day-old maize seedlings. Disease symptoms were rated 8 dpi. n represents the total number of inoculated plants from three independent experiments. **(B)** ER stress assay of *U*. *maydis* strain SG200 (WT) and derivatives. Serial 10-fold dilutions were spotted on YNBG solid medium supplemented with TM (0.5 μg/mL) as indicated. Plates were incubated for 48 h at 28°C.

SPPs have been implicated in ERAD-dependent processes in various organisms, including fungi, protozoa and mammals [28, 30, 31]. To test if the virulence function of Spp1 might be connected to ERAD-dependent protein degradation we deleted individually and in combinations the genes encoding the conserved ERAD components Hrd1 (*UMAG_00542*, ubiquitin-protein ligase), Doa10 (*UMAG_10911*, ubiquitin-protein ligase), Der1 (*UMAG_05898*, derlin-like) and Der2 (*UMAG_11402*, derlin-like) in the solopathogenic SG200 (WT) background. Single deletions of genes encoding Hrd1 and Der1 were already generated in the course of the UPR core gene deletion screen. Surprisingly, neither ER stress resistance nor virulence was affected in any of the single, double or triple mutants (Fig 8). This implies that ERAD does not play a major role in virulence of *U*. *maydis* and that the virulence function of Spp1 is not connected to ERAD-mediated protein degradation.

In *A*. *nidulans* and the human opportunistic pathogen *A*. *fumigatus* the SPP SppA mediates cleavage of the basic helix loop helix transcription factor SrbA (sterol regulatory element binding protein) under hypoxic conditions [33]. We speculated that the single protein related to SrbA in *U*. *maydis* (UMAG_05721/Srb1, E = 2.0e-14) might potentially be involved in adaptation to reduced oxygen levels *in planta*. To this end, we generated *srb1* deletion mutants in the solopathogenic strain SG200 (WT), but did not observe altered ER stress resistance or virulence of Δ*srb1* mutants in comparison to the WT control (S10 Fig). These results suggest that the virulence function of Spp1 is not related to Srb1-dependent processes. In human embryonic kidney cells (HEK293) the heme oxygenase 1 (HO1) is subject to SPP-dependent cleavage under hypoxic conditions [60]. To test whether *UMAG_00783*, the single gene predicted to encode a heme oxygenase in the *U*. *maydis* genome, is important for virulence we generated deletion mutants in the SG200 strain (WT). However, plant infection experiments revealed that deletion of *UMAG_00783* did not affect virulence when compared to the WT control (S11 Fig), suggesting that the virulence defect of Spp1 mutants is not related to the function of UMAG_00783.

### Spp1 is dispensable for effector secretion but required for suppression of plant defense responses

A critical step in the infection process of biotrophic pathogens is the establishment of a compatible interaction, which is mediated by secreted effector proteins. To test if *spp1* mutants are impaired in effector secretion we deleted *spp1* in the strain SG200Δ*pit2*-*P*_*otef*_:*pit2-mCherry* [46], expressing the Pit2-mCherry fusion protein under the control of the constitutive *otef* promoter. As additional control we used strain SG200Δ*pit2*-*P*_*otef*_:*pit2-mCherry*Δ*cib1*, which is defective in secretion of the Pit2-mCherry under ER stress conditions [46]. Secretion of Pit2-mCherry was visualized by Western hybridization 4 hours after TM-mediated ER stress induction. In the Δ*cib1* mutant, secretion of Pit2-mCherry was strongly reduced under UPR-inducing ER stress conditions, but not in the WT control or the Δ*spp1* mutant (S12 Fig). We employed the same strategy to test for altered secretion of the effectors Pep1 [15], Tin2 [19] and Cmu1 [20], expressed as mCherry (mC) fusion proteins in the Δ*spp1* mutant in comparison to the WT control. Consistent with the results obtained for Pit2-mC we did not observe obvious differences in effector secretion between WT and the Δ*spp1* mutant (S13 Fig). Thus, our data suggests that Spp1 is dispensable for effector secretion in axenic culture, Notably, under the conditions tested secretion of Pep1-mC and Tin2-mC was strictly dependent on TM-induced ER stress, whereas secretion of Cmu1-mC was not. Moreover, Pep1-mC and Cmu1-mC showed altered migration patterns, when cells were incubated under ER stress inducing conditions in comparison to the untreated control (S13 Fig), indicating that these proteins are selectively processed under these conditions. In summary, this suggests that induction of the UPR, but not expression of Spp1, might be critical for secretion and/or processing of a subset of effectors.

We reasoned that although Spp1 is not required for secretion of effectors under axenic conditions, Spp1 might be important to cope with the parallel expression and high amounts of effector proteins during establishment of the biotrophic interaction (second and strongest effector wave at 2 dpi) [22]. As this cannot be directly investigated, we used an indirect assay to address this assumption. If our assumption were correct, we would expect strongly elevated ER stress levels in the Δ*spp1* mutant at this stage. Hence, we used the expression levels of *cib1*^*s*^ and the fungal UPR marker genes *bip1*, *lhs1*, *cne1* and *UMAG_11594* as a read out, to compare ER stress levels in plants inoculated with Δ*spp1* and the *spp1*^*D279A*^ strains relative to the WT control 2 dpi. Expression of all UPR marker genes was similar in all strains (S14 Fig), suggesting that *spp1* deletion mutants do not suffer from increased ER stress during pathogenic development of *U*. *maydis*. In addition, this implicates that Δ*spp1* mutants are not impaired in effector secretion at this stage of biotrophic growth.

**Fig 9:**
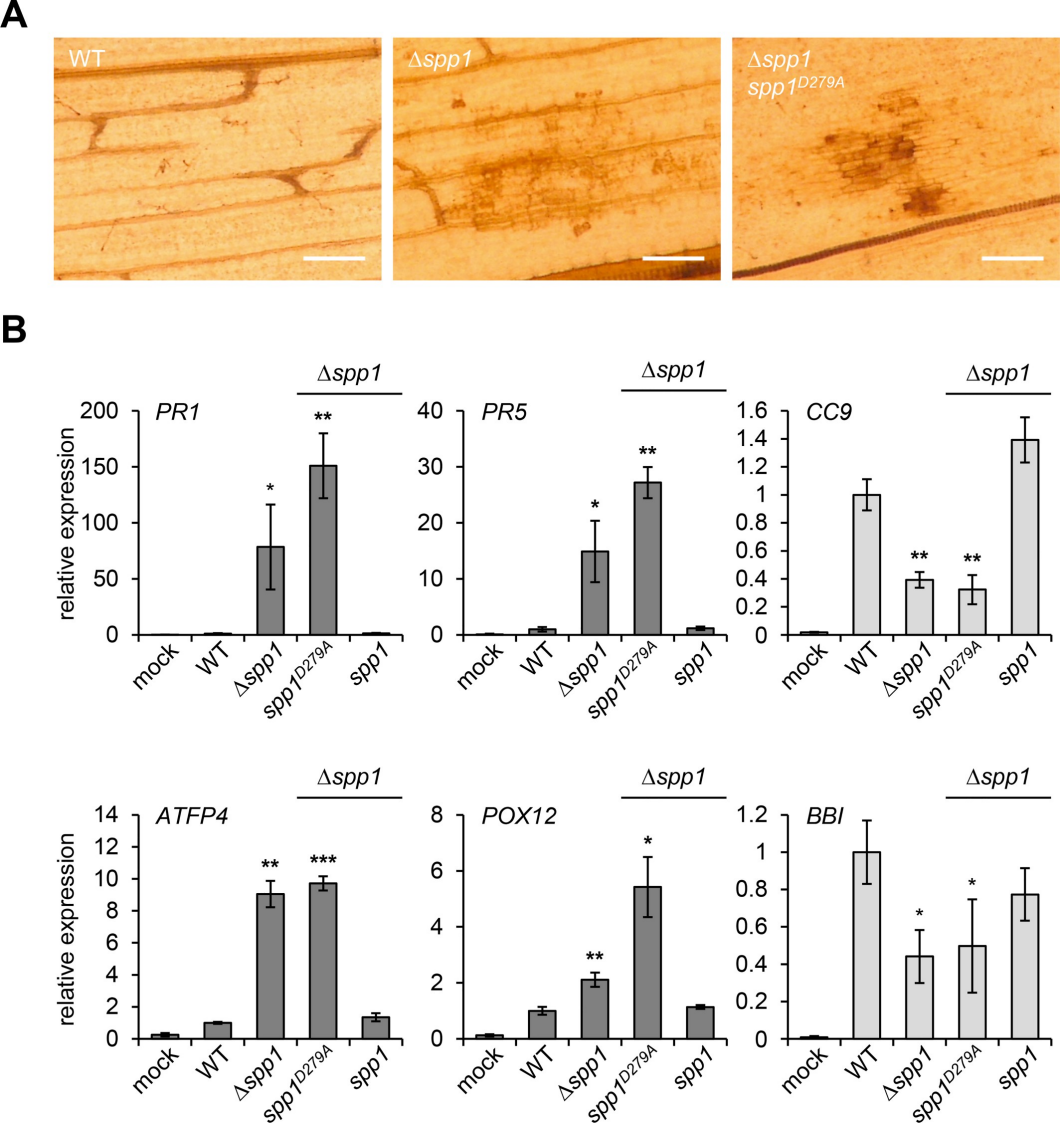
Spp1 is required for suppression of plant defense responses. **(A)** 3,3'-Diaminobenzidine (DAB) staining of leaf tissue infected with *U*. *maydis* SG200 (WT), SG200Δ*spp1* (Δ*spp1*) and SG200Δ*spp1*-*spp1*^*D279A*^ *(spp1*^*D279A*^*)* was performed 24 h post inoculation. Brown precipitates reflect the presence of reactive oxygen species (ROS). Scale bar = 100 μm. **(B)** qRT-PCR based expression analysis of defense related *Z*. *mays* genes in response to infection with indicated *U*. *maydis* strains. 7 day-old maize seedlings were used for inoculation and samples of infected leaf tissue were collected 2 dpi. Expression levels are depicted relative to plants infected with the WT and represent the mean of three biological replicates with two technical duplicates each. GAPDH was used for normalization. Dark grey and light grey color of the bars indicate SA responsive genes (*PR1*, *PR5*, *ATFP4*, *POX12*) and JA responsive genes (*CC9*, *BBI*), respectively. Error bars represent the SD. Statistical significance was calculated using Student’s *t* test. *P value ≤ 0.05, **P value ≤ 0.01 and ***P value ≤ 0.001.

Since Δ*spp1* mutants displayed problems during plant cell traversal (Fig 6), we wondered whether this might be caused by elevated plant defense responses. The failure to establish a compatible host-pathogen interaction results in the formation of plant derived reactive oxygen species (ROS) to counter plant invasion by the pathogen. We visualized ROS formation by 3,3’-diaminobenzidine (DAB) staining of leaf tissue derived from plants inoculated with the Δ*spp1* mutant, the *spp1*^*D279A*^ expressing strain and the SG200 (WT) control. Surprisingly, Δ*spp1* mutants showed not only local accumulation of DAB, but large areas of DAB precipitates (Fig 9A), indicative for the formation of ROS and the induction of a hypersensitive response. These precipitates were absent in tissue infected with the SG200 (WT) strain, and were slightly more increased in strains expressing the catalytically inactive Spp1^D279A^ instead of wildtype Spp1 (Fig 9A). We next asked whether Δ*spp1* mutant strains might be hypersensitive towards ROS. To this end, we performed drop plate assays using different concentrations of H_2_O_2_, but did not observe any differences between the WT, Δ*spp1* and Δ*spp1-spp1* complementation strains (S15A Fig). In addition, we performed plant infection experiments using the NADPH oxidase inhibitor Diphenyleneiodonium (DPI) as a supplement to counter plant-derived ROS formation as described previously [61, 62]. However, DPI treatment did not restore virulence or hyphal morphology of Δ*spp1* mutant strains *in planta* (S15B and S15C Fig). Overall, these data suggest that the loss of virulence in Δ*spp1* mutant strains cannot be attributed to hypersensitivity against ROS.

To assess the plant defense responses elicited by Δ*spp1* strains in a quantitative manner we analyzed expression of defense related plant genes by qRT-PCR analysis. As a read-out for salicylic acid (SA)-related defense responses we used the classical marker genes *PR1* and *PR5*, as well as *ATFP4* encoding a SA-induced metal-binding protein, *POX12* encoding an apoplastic peroxidase triggering ROS generation in response to pathogen attack as well as PR3 and PR4 (Fig 9B, and S16 Fig, dark grey bars.). For jasmonic acid (JA)-related responses we used *CC9*, encoding the cysteine protease inhibitor cystatin and *BBI* encoding the bax-inhibitor protein 1 as marker genes (Fig 9B, light grey bars). Typically, biotrophic pathogens attempt to suppress SA and induce JA-related defense responses to prevent programmed plant cell death [63]. Strikingly, expression of all SA marker genes was strongly induced in plants infected by Δ*spp1* mutants or the Spp1^D279A^ strain in comparison to plants infected by the SG200 (WT) or the *spp1*-complementation strain (Fig 9B and S16 Fig). The highest induction was observed for *PR1* with 78-fold and 151-fold increased levels in plants infected with Δ*spp1* or the *spp1*^*D279A*^ expressing strain in comparison to the WT control, respectively. By contrast, expression of both JA marker genes was significantly reduced in plants infected with the Δ*spp1* mutant or the *spp1*^*D279A*^ strain (Fig 9B). Hence, the catalytic activity of Spp1 is crucial for the suppression of SA-related plant defense responses, such as ROS generation and the hypersensitive response, revealing an unexpected function for Spp1 that is required for the establishment of a compatible interaction between a biotrophic fungus and its host.

In summary our study exploited Clp1-mediated UPR modulation to identify Spp1 as a novel UPR regulated virulence factor that is functionally conserved in related plant pathogens and higher eukaryotes. Since the enzymatic activity of Spp1 is essential for virulence, generation of cleavage products is essential for interference with plant defense responses. The discovery of SPP-like proteins as an inducible platform for suppression of plant defense responses by a fungal pathogen identifies a novel physiological role for SPPs and highlights how fungal pathogens adapt conserved pathways for specialized functions in host pathogen interactions.

## Discussion

In this study, we identified a set of 65 UPR core genes by RNAseq based transcriptome analysis. Modification of Cib1 functionality is based on Clp1-dependent posttranscriptional effects including reduced phosphorylation and increased stability of Cib1. Expression of UPR core genes is differentially affected, and not related to an altered DNA-binding specificity of Cib1. The UPR regulated signal peptide peptidase Spp1 represents a novel virulence factor that is crucial for plant defense suppression and the establishment of a biotrophic interaction.

In line with previous transcriptome analysis of the UPR in budding yeast and other filamentous fungi, the predicted gene functions of most UPR core genes identified in this study involve ER associated process (Fig 1C) important for the adaptation of the secretory pathway during ER stress [25, 26, 64, 65]. A direct regulation of the majority of UPR core genes by Cib1 was deduced from ChIPseq analysis. Hac1-like proteins are highly divergent with respect to their amino acid sequence, but conserved in their bZIP domain and function and are thus expected to bind similar UPREs in different species [66]. Here, we identified an UPRE in the promoters of Cib1-regulated UPR core genes that closely resembles the binding site of CRE3 and the Hac1-homolog XBP1 in higher eukaryotes [52, 53] (Fig 3A). Based on the functional complementation of Δ*hac1* strains by expression of the spliced *cib1*^*s*^ mRNA, we previously used Hac1 DNA binding data [67] to predict UPREs in the promoter region of effector encoding genes in the *U*. *maydis* genome [46]. Validation of Cib1 binding to *tin1-1* and *pit1/2* promoter regions by ChIP-qPCR suggested a Cib1 binding site that is highly similar to the UPRE identified by genome wide ChIPseq analysis. However, in the current study we only observed differential gene expression for *pit1* and reproducible binding to the *tin1-1* promoter (S1 Table and S3 Fig), suggesting that this might result from the different strain backgrounds used (SG200 vs. JB1). The solopathogenic SG200 strain harbors an active *b*-pathway that is inactive in JB1 due to the full deletion of the *b*-mating type locus [11]. As the *b*-dependently expressed transcription factor Hdp2 is involved in the regulation of *pit1/2* expression [68], it appears possible that binding of Cib1 to the *pit1/2* promoter might involve Hdp2.

Modulation of the UPR is observed in diverse organisms, and a logical consequence of the multitude of interacting pathways and different lifestyles of individual species [43, 45, 69–73]. Modulation of the UPR can occur upstream of transcriptional regulation by regulation of Ire1-phosphorylation [74], membrane alterations [75], iron abundance [76], as well as downstream by modulation of HAC1 mRNA or protein stability [77–79]. The increased complexity of the UPR in higher eukaryotes, in which besides the evolutionary conserved Ire1/Hac1(XBP1) axis, two additional arms of the UPR regulated by the bZIP transcription factors ATF6 and ATF4 exist, provides additional means of UPR modulation. ATF6 transcriptionally activates expression of XBP1 [80] and forms heterodimeric transcription factor complexes with XBP1 [81], resulting in regulation of overlapping and specific subsets of target genes by the individual complexes [82]. ATF4 is the homolog of the central regulator of general amino-acid control Gcn4 in budding yeast, and is involved in execution of cell death if ER stress cannot be resolved [24]. Although both pathways are mutually interconnected, the anticipated physical interaction between Gcn4 and Hac1 has not been demonstrated [83, 84].

The reduced phosphorylation of Cib1 in Clp1 expressing strains correlated with increased protein stability and strongly elevated ER stress resistance [36] (Figs 1A and 4D). In budding yeast, Hac1 and Gcn4 are phosphorylated during transcription initiation by the Srb10 kinase, a component of the SRB/mediator module of RNA polymerase II [78, 85–88]. Phosphorylation targets the bZIP proteins for SCF^Cdc4^-dependent ubiquitylation and proteasomal degradation, generating a negative feedback loop referred to as the "black widow" model [87].

Stabilization of Hac1 by deletion of *SRB10* or *CDC4*, or by mutation of the phospho-sites targeted by Srb10, resulted in increased ER stress resistance [78]. In contrast to yeast, where stabilization of Hac1 led to increased expression of the UPRE reporter, Clp1-mediated stabilization of Cib1 resulted in a more complex regulation of UPR gene expression including repression of a subset of UPR core genes. This suggests, that although the underlying principle and effects on ER stress resistance might be similar between yeast and *U*. *maydis*, the consequences on gene regulation are different.

Although our localization studies revealed an altered subcellular localization pattern of Cib1-GFP when *clp1* is co-expressed, it remains to be determined whether this is directly related to the modulation of Cib1 function. Our ChIPseq data do not provide evidence for alterations of the Cib1-binding specificity that might account for the Clp1-dependent effects on UPR gene expression. However, we observed that the most strongly regulated UPR core genes showed higher promoter scores in JB1 (WT) in comparison to the UVO151 (+*clp1*) strain (Fig 3C). In *N*. *crassa*, activity of the white-collar complex (WCC), involved in the light-dependent regulation of the circadian clock, correlates with reduced WCC stability that is triggered by DNA-binding and counteracted by frequency-dependent nuclear exclusion [89, 90]. Thus, it is well possible that a similar mode of action applies to the Cib1/Clp1 interaction.

In conclusion, our results support a hypothetical model in which Clp1 negatively affects transcriptional activity of Cib1, leading to reduced phosphorylation and increased stability of Cib1. To this end, binding of Clp1 might either i) mask the Cib1 transactivation domain, ii) interfere with Cib1 homodimer formation or iii) alter the subcellular localization of Cib1.

The estimated half-life of Clp1 (t_1/2_<30 minutes) [36] is much shorter than of Cib1 (t_1/2_>60 minutes) (Fig 4D and S1 Fig). Accordingly, this inhibitory effect would be transient, thereby preventing excessive effects on UPR gene expression. Clp1 and Cib1 are expressed throughout all stages of fungal development *in planta* [8, 22, 36], suggesting that the UPR is continuously modulated. In this respect, Clp1 could buffer fluctuating demands on the secretory pathway e.g. as imposed by effector waves and maintain stable UPR gene expression during the different stages of biotrophic development.

RNAseq analysis revealed that factors previously shown to be important for ER stress resistance in *U*. *maydis* or other fungi such as the co-chaperone Dnj1 (UMAG_05173) [49], the protein disulfide isomerase Pdi1 (UMAG_10156) [48], the ER oxidoreductase Ero1 (UMAG_05219) [91] and the ER chaperones Lhs1 (UMAG_00904) [47] and Bip1 (UMAG_15034) [92], showed reduced expression levels during *clp1*-modulated UPR. With the aim to identify novel factors for ER stress resistance and virulence, UPR core genes were chosen for gene deletion based on increased or unchanged expression levels during *clp1*-modulated UPR. This set of genes comprised mainly factors that were previously not connected to any of these functions. To our surprise, we identified not a single gene important for ER stress resistance (S4 Fig). Thus, contrary to our expectations, in this group of 29 UPR core genes no enrichment of genes involved in either ER stress resistance or virulence was observed. This finding might be explained by the existence of proteins with redundant or partially overlapping functions, which might support the robustness of the pathway [93] or help in fine-tuning and adapting the pathway during changing environmental conditions *in planta*. Alternatively, the physiological role of *clp1*-dependent UPR modulation might be primarily to prevent the deleterious overexpression of the above-mentioned known UPR genes, as previously hypothesized [36].

Deletion of *spp1* did not affect ER stress resistance but completely abolished virulence (S5 Fig), suggesting that Spp1 is a key factor for fungal virulence. Spp1 is specifically required during biotrophic growth since neither vegetative growth, nor resistance to ER- or cell wall stress was affected in *spp1* deletion strains. The conserved domain architecture and catalytic YD/GLGD and substrate binding (QPALLY) motifs, as well as the subcellular localization pattern and the observation that deletion of *spp1* can be complemented with the human SPP HM13, strongly suggests that Spp1 is a bona fide SPP, cleaving type 2 transmembrane domains. The catalytic activity of Spp1 is required for its virulence specific function, since expression of Spp1^D279A^ harboring a mutation of the conserved aspartate in the catalytic YD/GLGD motif [29, 94] did not rescue the virulence defect of *spp1* deletion strains. Spp1 is the single SPP encoded in the *U*. *maydis* genome, arguing against the possibility that functionally redundant proteins encoded in the *U*. *maydis* genome may mask specific functions of Spp1 during normal growth or stress adaptation. Although SPP proteins are implicated in ER homeostasis [28, 30], transcriptional regulation of SPPs by the UPR has not been reported for other organisms. However, we observed strong conservation of the Cib1 binding site and its location in the *spp1* promoter of related smut species (S17 Fig). Together with the increased expression of *spp1* orthologs during biotrophic growth of *U*. *hordei* and *Ustilago bromivora* [95, 96], this suggests that the connection between the UPR, Spp1 and fungal virulence might not be restricted to *U*. *maydis*.

It is tempting to speculate that in *U*. *maydis* cleavage of specific SPP substrates generates products with virulence specific functions. SPP substrates include but are not limited to subsets of signal sequence remnants in the ER membrane after processing of the precursor protein by the signal peptidase complex (SP) and type II transmembrane proteins [28, 31, 97, 98]. Most substrates are subsequently degraded and pharmacological inhibition of the SPP activity in *P*. *falciparum* interferes with ERAD and prevents intraerythrocytic development [99]. In budding yeast and humans, SPP cleavage of the high-affinity zinc transporter Zrt1 and the UPR repressor XBP1u, respectively, targets the cleaved proteins for degradation via ERAD as well [28, 31]. Since ERAD is not of major importance for ER stress resistance and virulence in *U*. *maydis*, the virulence phenotype of *spp1* deletion strains is most likely not connected to this pathway. In the human pathogen *A*. *fumigatus*, only multiple deletions of genes encoding ERAD components led to reduced ER stress resistance but did not affect pathogenicity [100]. Importantly, SPP cleavage products are not always degraded but also exert biological functions. In humans, SPP cleavage of the hepatitis core protein C promotes trafficking of the core protein to lipid droplets [97], and processing of MHC class I signal sequences generates cell surface epitopes that protect against the attack of natural killer cells [32]. However, similar mechanisms do not exist in fungi or plants and a major function for hypoxia adaptation through Spp1-mediated cleavage of the SREBP transcription factor appears unlikely.

The establishment of a compatible interaction between biotrophic pathogens and their host requires plant defense suppression by secreted effectors [14]. However, Spp1 is not important for protein secretion as evidenced by monitoring secretion of Pit2, Pep1, Tin2 and Cmu1 under axenic conditions or expression of UPR marker genes during biotrophic development, contrasting the central role of a functional UPR for efficient secretion and processing of effector proteins [36, 46, 49]. Remarkably, secretion of Pep1 and Tin2 and processing of Pep1 and Cmu1 is strictly dependent on ER stress, providing obvious clues on how effector secretion and function might be coordinated and potentially modulated by the UPR and altered ER stress levels.

Since *U*. *maydis* depends on the conserved catalytic activity of Spp1 but not on ERAD or other previously identified SPP substrates to cause disease we postulate that plant defense suppression is either achieved by specific activation of Spp1, or, more likely, by cleavage of substrates that are specifically expressed during the biotrophic stage. Collectively, our data revealed a novel pathway of a biotrophic pathogen to suppress the plant defense and establish a compatible host-pathogen interaction.

## Materials and methods

### Strains and growth conditions

*Escherichia coli* strain TOP10 (Invitrogen) was used for cloning purposes and amplification of plasmid DNA. *Ustilago maydis* cells were grown at 28°C in yeast-extract-peptone-sucrose (YEPS) light medium [101], complete medium (CM) [102], or yeast nitrogen base (YNB) medium [103], supplemented with 1% glucose or 1% arabinose. Induction of P_*crg1*_ driven gene expression was performed as described by [104]. Filamentous growth was induced on potato dextrose plates containing 1% (w/v) activated charcoal [102]. ER stress tolerance was tested on yeast-nitrogen-base (YNB) media containing the indicated concentrations of tunicamycin (Sigma-Aldrich). Sensitivity towards H_2_O_2_, congo-red or calcofluor white was tested in drop-plate assays on YNB solid media containing the indicated concentrations of respective supplements. *U*. *maydis* strains used in this study are listed in (S5 Table).

### DNA and RNA procedures

Molecular methods used in this study followed described protocols [105]. DNA isolation and transformation procedures for *U*. *maydis* were performed as described previously [9]. Linearized plasmids or PCR amplified DNA was used for homologous integration into the *U*. *maydis* genome. All constructs were verified by Sanger sequencing prior transformation or PCR amplification. Correct integration of constructs into the *U*. *maydis* genome was verified by Southern hybridization. Q5 polymerase (NEB) was used for PCR amplification.

Primers used in this study can be found in (S6 Table). RNA was prepared from cells during exponential growth in axenic culture or from infected maize plants using Trizol reagent (Invitrogen) according to the manufacturer’s instructions [11], followed by removal of residual DNA with Turbo DNase (Ambion/Lifetechnologies). If RNA was used for RNAseq analysis samples were further column-purified with the RNeasy Kit (Qiagen). Integrity of RNA was checked by ethidium bromide staining or with a Bioanalyzer equipped with an RNA 6000 Nano LabChip kit (Agilent). All gene deletions were performed using a PCR based approach [106].

### Plasmid constructions

For the *cib1-GFP* fusion, the 5.5 kb *SfiI* 3xGFP-HygR fragment of plasmid pcib1-3xGFP [8] was replaced with the 2.5 kb *SfiI GFP-Nat*^*R*^ fragment from pUMa389 [107] to generate the plasmid *pcib1-GFP*. The resulting vector was used to generate plasmid pcib1-3xHA by exchanging the *SfiI* GFP-Nat^R^ cassette with a 1.8 kb *SfiI* 3xHA-Nat^R^ fragment from pUMa793 [108].

For replacement of the *cib1* promoter with a tetracycline-regulated promoter, 1kb upstream of the *cib1* start codon and 1kb of the *cib1* open reading frame (ORF) were PCR amplified from genomic DNA, ligated to the *SfiI* cassette of pUMa707 [54] and integrated in the pCR2.1 TOPO vector (Invitrogen) generating plasmid pP_tef-tTA-tetO_:cib1. To generate the *spp1-mCherry* fusion, the ORF of *spp1* (*UMAG_02729*, UM521) lacking the stop codon was PCR amplified from genomic DNA introducing a *BamHI* site at the 5’ end and a *BspHI* site at the 3’ end and integrated into p123-mCherry [109], to yield p123:spp1-mCherry. Cloning of orthologous genes from *Sporisorium reilianum Srspp1* (*sr13785*, strain SRZ1), *Ustilago hordei Uhspp1* (*UHOR_04354*, strain Uh4857-4) and *Aspergillus nidulans sppA* (ANID_08681, strain AGB551) followed the same procedure, generating plasmids p123:Srspp1-mCherry, p123:Uhspp1-mCherry and p123:sppA-mCherry, respectively.

For cloning of *S*. *cerevisiae YPF1* the ORF (YKL100C, strain sigma 1287) was PCR amplified from genomic DNA introducing *BamHI* sites at the 5’ and 3’ end removing the stop codon, and integrated into p123-mCherry to yield plasmid p123:YPF1-mCherry.

The cDNA of the human *HM13* (*BC062595*, cDNA clone) was PCR amplified from the vector *pCS6(BC062595)-TCH1303-GVO-TRI* (BioCat) introducing a *XmaI* site at the 5’ end, a *NcoI* site at the 3’ end and removing the stop codon and subsequently ligated into p123-mCherry to yield p123:HM13-mCherry. To replace the *otef* promoter in p123:spp1-mCherry with the *spp1*-promoter, a 1.4kb *spp1* promoter fragment was PCR amplified introducing a *NdeI* site at the 5’ end and a *BamHI* site at the 3’ end. The PCR fragment was integrated into p123:spp1-mCherry to generate pP_spp1_:spp1-mCherry. To generate the catalytically inactive version of *spp1 (spp1*^*D279A*^), a point mutation was introduced into the ORF of *spp1* by standard PCR procedures. Cloning of the PCR fragment followed the procedure as described for p123-spp1:mCherry, yielding plasmid p123:spp1^D279A^-mCherry. To generate the *pep1-mCherry* fusion, the ORF of *pep1* (*UMAG_01987*, UM521) lacking the stop codon was PCR amplified from genomic DNA introducing a *BamHI* site at the 5’ end and a *NcoI* site at the 3’ end and integrated into p123-mCherry [109], to yield p123:pep1-mCherry. To generate the *tin2-mCherry* fusion, the ORF of *tin2* (*UMAG_05302*, UM521) lacking the stop codon was PCR amplified from genomic DNA introducing a *BamHI* site at the 5’ end and a *NcoI* site at the 3’ end and integrated into p123-mCherry [109], to yield p123:tin2-mCherry. To generate the *cmu1-mCherry* fusion, the ORF of *cmu1* (*UMAG_05731*, UM521) lacking the stop codon was PCR amplified from genomic DNA introducing a *XmaI* site at the 5’ end and a *NcoI* site at the 3’ end and integrated into p123-mCherry [109], to yield p123:cmu1-mCherry.

### Microscopy

Microscopic analysis was performed using an Axio Imager.M2 equipped with an AxioCam MRm camera (ZEISS) or an Axio Imager.M1 (ZEISS) equipped with a CoolSNAP HQ2 CCD camera (PHOTOMETRICS). All images were processed with ZEN 2.3 blue edition (ZEISS).

Chlorazol Black E staining was performed according to [104]. For microscopic analysis of cells after TM treatment *U*. *maydis* strains were grown in CM to an OD_600_ of 0.35. TM was added to a final concentration of 5 μg/ml and cells were incubated for the indicated time to induce the UPR.

For detection of reactive oxygen species (ROS) in infected leaf tissue, 3,3’-diaminobenzidine (DAB) was used as described previously [61]. Briefly, leaves (third leaf) were detached with a razor blade 1 cm above and 2 cm below the injection site 24h post infection and incubated for 12h in 1 mg/ml DAB solution under darkness at room temperature. For decolorization, leaves were immersed in ethanol (96%) for 48h. For storage of the specimens, the leaves were transferred into 10% (v/v) glycerol. Brown polymerization products resulting from the reaction of DAB with ROS were microscopically identified using a binocular microscope (Keyence Digital Microscope VHX-500F).

### Quantitative RT-PCR (qRT-PCR) analysis

qRT-PCR analysis was carried out as described before [46]. For all qRT-PCR experiments, mRNA was isolated from three independent biological samples, subjected to cDNA synthesis (RevertAid First Strand cDNA Synthesis Kit, Thermo Scientific) and analyzed in two technical repeats using MESA Green qPCR Mastermix Plus (Eurogentec). qRT-PCR was performed on CFX Connect Real-Time PCR Detection System and the CFX Manager Software (BioRad). Statistical significance was calculated with Student’s *t*-test.

### RNA sequencing (RNAseq)

For RNAseq, strains were grown in YNB supplemented with 1% glucose and 0.2% ammonium sulfate (YNBG) overnight to an OD_600_ of 0.25 and shifted to YNB supplemented with 1% arabinose and 0.2% ammonium sulfate (YNBA) to induce Clp1 expression (*P*_*crg1*_:*clp1*). To induced the UPR, TM was added to a final concentration of 5 μg/ml and cells were further incubated for 4 hours at 28°C. Cells were harvested and quick-frozen in liquid nitrogen. RNA extraction followed the procedure as described above.

5 μg of total RNA was used to enrich mRNA using the NEB Next Poly(A) mRNA Magnetic Isolation Module (NEB) according to the manufacturers instructions. Strand-specific cDNA libraries were constructed with the NEBNext Ultra directional RNA library preparation kit for Illumina (NEB). To assess quality and size of the libraries samples were run on an Agilent Bioanalyzer 2100 using an Agilent High Sensitivity DNA Kit as recommended by the manufacturer (Agilent Technologies). Concentration of the libraries was determined using the Qubit dsDNA HS Assay Kit as recommended by the manufacturer (Life Technologies). Sequencing was performed using the HiSeq4000 instrument (Illumina Inc) and the HiSeq 3000/4000 SR Cluster Kit for cluster generation and the HiSeq 3000/4000 SBS Kit (50 cycles) for sequencing in the single-end mode, running 1x 50 cycles. A minimum of 15 Million raw reads were generated for individual samples.

Raw RNAseq reads were aligned to the *Ustilago maydis* genome from Ensembl Genomes 33 [110] using STAR [111] version 2.4.1. Read counts and RPM (reads per million) were calculated using custom Python scripts. Differential expression was assessed with DESeq2 [112] at an FDR threshold of 0.05 and a log2 fold change threshold of 1 or 2. RNAseq data was deposited at EBI ArrayExpress (https://www.ebi.ac.uk/arrayexpress/) under accession E-MTAB-7463.

Heat map of identified UPR core genes was visualized using ClustVis Web Tool [113]. Hierarchical clustering was performed using Euclidean distance and complete linkage for genes. UPR core genes were further analyzed using the Functional Catalogue annotation of the MIPS *U*. *maydis* database (http://mips.gsf.de/funcatDB/).

### Chromatin immunoprecipitation sequencing (ChIPseq)

ChIP analysis was done essentially as described before [46], with the modification that chromatin was sheared in a Covaris S200 set to yield a DNA average size of approximately 100–300 bp. DNA was recovered by column purification (PCR Purification Kit, Qiagen) and subjected to library preparation.

For ChIPseq experiments the libraries were prepared from 1 ng of enriched DNA or input DNA using the NEBNext Ultra II DNA Library Prep with Beads as recommended by the manufacturer (New England BioLabs). To assess quality and size of the libraries, samples were run on an Agilent Bioanalyzer 2100 using an Agilent High Sensitivity DNA Kit as recommended by the manufacturer (Agilent Technologies). Concentration of the libraries was determined using the Qubit dsDNA HS Assay Kit as recommended by the manufacturer (Life Technologies GmbH). Libraries were sequenced on a HiSeq4000 instrument (Illumina Inc) using the HiSeq 3000/4000 SR Cluster Kit for cluster generation and the HiSeq 3000/4000 SBS Kit (50 cycles) for sequencing in the single-end mode, running 1x 50 cycles. A minimum of 40 Million raw reads were generated for the ChIPseq experiments.

Raw ChIPseq reads were aligned using Bowtie2 [114] version 2.0.0-beta7 to the *Ustilago maydis* genome from Ensembl Genomes 33 [110]. Peak calling was performed using PeakZilla [51], GitHub commit version 7167f084e024676bcb34e5b5c3e1281910423c25. ChIPseq data was deposited at EBI ArrayExpress (https://www.ebi.ac.uk/arrayexpress/) under accession E-MTAB-7460. Peak calling was performed individually for both biological replicates. Only peaks identified in both replicates were used for further analyses. Assignment of peaks to genes in case of divergent promoters was based on the relative distance to the translational start site (tss) and on gene expression after TM treatment. Peak scores were accumulated to promoter scores if more than one peak was identified in the promoter of a single gene and at least one peak score was above 40. Promoter scores were filtered by a cut-off of 100. Promoters harboring more than four peaks could never be assigned to differentially expressed genes and were thus discarded from further analysis. Normalized bigWig files were generated from BAM files derived from both replicates and visualized using the Integrative Genomics Viewer (IGV) [115]. For identification of possible binding motifs of Cib1, sequences of assigned ChIP peaks derived from the UPR core gene set were subjected to the MEME (Multiple EM for Motif Elicitation)-ChIP analysis [116].

### Plant infection studies

For plant infection studies the maize (*Zea mays*) cultivar Early Golden Bantam was used under controlled conditions using a CLF Plant Climatics GroBank with a 14 h (28°C)/10 h (22°C) day/night cycle. *U*. *maydis* strains were incubated in YEPS_light_ at 28°C to a final OD_600_ of 0.8–1.0, washed with H_2_O and concentrated to an OD_600_ of 1.0 in H_2_O. 300–500 μl of the cell suspension were injected into the basal stem of 7 day-old maize seedlings. DPI treatment was performed as described previously [61, 62]. All infection experiments were repeated three times or as otherwise indicated. Disease rating was performed according to [56].

### Protein procedures

Protein isolation and Western hybridization experiments were performed as described previously [117]. Commercially available rabbit anti-GFP (Sigma-Aldrich) (1:4000 dilution) or anti-RFP [6G6] (Chromotek) (1:1000 dilution), were used to detect GFP- or mCherry-fusion proteins, respectively. Horseradish peroxidase-conjugated anti-mouse IgG (Promega) were used as secondary antibody. The Luminata Crescendo Western HRP substrate (Merck Millipore) was used for chemiluminescence detection.

Secretion assays of Pit2-mC, Pep1, Tin2 and Cmu1 were performed as described previously [46], with small alterations. Instead of DTT, TM (5μg/ml) was used to induce ER stress for 4 h prior to protein preparation. Stability of Cib1-GFP in response to Clp1 expression was determined with a doxycycline (DOX) based promoter shut-off system (P_tetO_:cib1-GFP) [54]. *U*. *maydis* strains were grown in CM supplemented with 1% glucose (CMG) to an OD_600_ of 0.35, and shifted to CM supplemented with 1% arabinose (CMA) to induce P_*crg1*_-driven *clp1* expression. For UPR activation, TM was added to a final concentration of 5 μg/ml. 4 h after UPR induction DOX (10 μg/ml) was added (T0) and protein extract was prepared from samples taken at the indicated time points [1 h (T1), 2 h (T2), 3 h (T3) and 4 h (T4)] after DOX treatment. Cycloheximide (CHX)-based determination of Cib1-GFP stability was performed as described before [36]. Briefly, cells were grown as described for promoter shut-off assays. Protein biosynthesis was inhibited using CHX (100 μg/ml) and cells were sampled directly before (T0), or at the indicated times after CHX treatment [30 min (T1), 60 min (T2) or 90 min (T3)]. Cib1-GFP levels ImageJ were quantified using (https://imagej.nih.gov/ij/) and normalized to Ponceau S stained bands.

Stability of proteins was calculated relative to T0. Experiments were performed in three biological replicates. Statistical significances (P value) were calculated using Student’s *t* test.

Protein phosphatase assays were performed after immunoprecipitation of Cib1-GFP followed by on-bead treatment with lambda-phosphatase (NEB). Cells were incubated as described for promoter shut-off experiments. Briefly, 4 hours after TM-mediated UPR activation (5 μg/ml) equal culture volumes were centrifuged, cell pellets were washed once with tris buffered saline (20 mM Tris-HCl, 137 mM NaCl, pH 7.6, supplemented with 2x cOmplete proteinase inhibitor (ROCHE) (PI) and phosphatase inhibitor cocktail (1 mM NaF, 0.5 mM Na_3_VO_4_, 8 mM β-glycerophosphat, PhI). The pellet was resuspended in 750 μl buffer B-300 (300 mM NaCl, 100 mM Tris, pH 7.5, 10% Glycerol, 1 mM EDTA, supplemented with 2x PI and PhI), shock frozen in liquid nitrogen and disrupted in a cell mill (Retsch MM400, 30Hz, 2min). After cell lysis, 750 μl of B+300 buffer (300 mM NaCl, 100 mM Tris (pH 7.5), 10% Glycerol, 1 mM EDTA, 0.1% NP40, supplemented with 2x PI and PhI) was added and the whole cell lysate was centrifuged at 45,000 rcf for 30 minutes at 4°C. The supernatant was added to 60 μl of magnetic agarose GFP-Trap beads (Chromotek) and incubated for 3h at 4°C on a rotating wheel. After washing the beads 2x with 500 μl of B-300 buffer and removing the supernatant, beads were resuspended in 600 μl of buffer B-300 (supplemented with 2x PI) and evenly distributed in 200 μl aliquots. The supernatant was discarded and 1x lambda phosphatase buffer (NEB) was added to each sample. 1200U of lambda phosphatase were added. Control samples were left untreated or supplemented with 2x PhI. After incubation for 30 minutes at 30°C the supernatant was discarded and 30 μl 1x Roti Load 1 (Carl-Roth) was added to the beads and boiled at 98°C for 3 min. Samples were run on a 10% SDS-PAGE and subjected to Western hybridization. All steps were performed in Protein LoBind Tubes (Eppendorf). Experiments were repeated at least three times.

### Accession numbers

Sequence data from this article can be found at the Munich Information Center for Protein Sequences *Ustilago maydis* database (http://mips.helmholtz-muenchen.de/genre/proj/ustilago/) and the National Center for Biotechnology Information database under the following accession numbers:

*UMAG_00258*, XP_011386180.1; *hrd1*, *UMAG_00542*, XP_011386378.1; *UMAG_00783*, XP_011386557.1; *lhs1*, *UMAG_00904*, XP_011386916.1; *UMAG_01025*, XP_011387002.1; *UMAG_01112*, XP_011387066.1; *UMAG_01232*, XP_011387166.1; *pit2*, *UMAG_01375*, XP_011387264.1; *pep1*, *UMAG_01987*, XP_011387901.1; *clp1*, *UMAG_02438*, XP_011388726.1; *UMAG_02487*, XP_011388764.1; *spp1*, *UMAG_02729*, XP_011389095.1; *UMAG_02944*, XP_011389351.1; *UMAG_03404*, XP_011389878.1; *UMAG_03507*, XP_011389952.1; *UMAG_03541*, XP_011389978.1; *UMAG_03665*, XP_011390151.1; *ost3*, *UMAG_04198*, XP_011390684.1; *UMAG_04605*, XP_011390905.1; *eIF2b*, UMAG_04869, XP_011391708.1; *UMAG_04896*, XP_011391221.1; *UMAG_05009*, XP_011391306.1; *tin2*, *UMAG_05302*, XP_011392015; *mpd1*, *UMAG_05352*, XP_011392054.1; *srb1*, *UMAG_05721*, XP_011391469.1; *cmu1*, *UMAG_05732*, XP_011391476.1; *der1*, *UMAG_05898*, XP_011392243.1; *UMAG_10006*, XP_011386596.1; *cln1*, *UMAG_10287*, XP_011389173.1; *UMAG_10686*, XP_011391738.1; *doa10*, *UMAG_10911*, XP_011390969.1; *UMAG_10921*, XP_011386693.1; *UMAG_11083*, XP_011390090.1; *UMAG_11190*, XP_011392333.1; *der2*, *UMAG_11402*, XP_011388858.1; *UMAG_11513*, XP_011390555.1; *UMAG_11594*, XP_011392502.1; *UMAG_11651*, XP_011387452.1; *UMAG_11763*, XP_011391003.1; *cib1*, *UMAG_11782*, XP_011390112.1; *UMAG_12149*, XP_011386842.1; *UMAG_12178*, XP_011388139.1; *UMAG_12304*, XP_011391965.1; *UMAG_12318*, XP_011392356.1; *UMAG_12332*, XP_011388414.1; *bip1*, *UMAG_15034*, XP_011387505.1; *Srspp1*, *sr13785*, CBQ73124.1; *Uhspp1*, *UHOR_04354*, CCF52970.1; *Ubspp1*, *UBRO_04354*, SAM82079.1; *HM13*, *BC062595*, NP_110416.1; *sppA*, *ANID_08681*, XP_681950.1; *sppA*, *AFUA_6G02150*, XP_747862.1; *YPF1*, *YKL100C*, NP_012822.1; *Pfspp*, *PF3D7_1457000*, XP_001348717.2; *SPPL2B*, NP_694533.1; *PR1*, AAC25629.1; *PR3*, NP_001340366.1; PR4, NP_001130495; *PR5*, NP_001105702.2; *CC9*, BN000513.1; *BBI*, EU955113.1; *POX12*, ACG36543.1; *ATFP4*, NP_001152411.1; *GAPDH*, NP_001105413.1

## Supporting information

S1 Fig Analysis of Cib1-GFP stability by cycloheximide (CHX) chase assay. Exponentially growing strains JB1*cib1-GFP* (WT) and UVO151*-cib1-GFP* (P_*crg*_:*clp1*) were shifted to CMA liquid medium supplemented with TM (5 μg/ml) to induce *clp1* and Cib1-GFP expression, respectively. Strains were further incubated for 4 h at 28°C, and 100 μg/ml CHX was added to inhibit protein expression. Protein extracts were prepared from samples taken directly before (T0) and 30 min (T1), 60 min (T2) or 90 min (T3) after CHX treatment and analyzed by Western hybridization with GFP specific antibodies. Ponceau S-stained membranes were used as loading control and for normalization of Cib1-GFP levels. Expression levels at T1, T2 and T3 were calculated relative to T0 using ImageJ. Values represent the mean of three biological replicates and error bars indicate the SEM. Statistical significance was calculated using Student’s *t* test. *P value ≤ 0.05.
(TIF)

S2 Fig ChIP q-PCR validation of data obtained by ChIPseq. ChIP q-PCR was performed on selected promoters of the corresponding genes *cib1* (*UMAG_11782*) and *ero1* (*UMAG_05219*) to test for promoter enrichment. The experiment was performed as described in Fig 3A. The gene *eIF2b* served as negative control. Enrichment was depicted relative to input DNA. Values represent the mean of three biological replicates and two technical duplicates each. Error bars indicate the SD. Statistical significance was calculated using Student’s *t* test. ***P value ≤ 0.001.
(TIF)

S3 Fig ChIPseq analysis of effector genes *pit1/2* and *tin1-1 in U. maydis*. Visualization of Cib1 binding to promoters of *U*. *maydis* effector genes *pit1* and *tin1-1* obtained by ChIPseq analysis. Strains, growth conditions and visualization of data was performed as described in Fig 3A.
(TIF)

S4 Fig ER stress resistance of UPR core gene deletion mutants. ER stress assay of *U*. *maydis* strain SG200 (WT) and derivatives. Serial 10-fold dilutions were spotted on YNBG solid medium supplemented with TM (0.5 μg/ml) as indicated. Plates were incubated for 48 h at 28°C.
(TIF)

S5 Fig Infection assay of UPR core gene deletion mutants. *U*. *maydis* strain SG200 (WT) and derivatives were inoculated into 7 day-old maize seedlings. Disease symptoms were rated 8 d after inoculation and grouped into categories depicted below. n represents the number of inoculated plants in a single infection experiment.
(TIF)

S6 Fig Multiple alignment of Spp1 homologs. Protein sequences of *U*. *maydis* Spp1 and predicted orthologs from indicated species were aligned using the MUSCLE algorithm (https://www.ebi.ac.uk/Tools/msa/muscle) and visualized by JalView (http://www.jalview.org). Full alignment is shown in **(A)**, and conserved sequence motifs are highlighted in **(B)**. The YD and GLGD motifs represent the active site of the aligned signal peptide peptidases. The QPALLY motif is conserved in all sequences except for *Aspergillus fumigatus*. Conservation of the sequence is shown on base of physico-chemical properties in the histogram below. + indicates that all properties are conserved (10) and values can go up to 11 (marked by *) in case of full conservation and identical amino acids. The Clustal X color scheme was used to group amino acids with similar properties.
(TIF)

S7 Fig Spp1-mC accumulation requires Cib1-dependent UPR. Localization and accumulation of the Spp1-mCherry fusion protein was monitored by fluorescence microscopy. Spp1-mC was expressed under the control of the native spp1- or the constitutive otef-promoter in WT and Δ*cib1* mutant background. Cells were analyzed 2 h after TM-mediated UPR induction and compared to the untreated control. Cellular morphology was visualized by DIC microscopy. Scale bars = 10 μm.
(TIF)

S8 Fig Analysis of cell wall stress resistance in Δ*spp1* strains. Cell wall stress resistance of *U*. *maydis* strain SG200 (WT) and the Δ*spp1* derivative was tested by serial 10-fold dilutions of strains, spotted on YNBG solid medium supplemented with Calcofluor White (50 μM) or Congo Red (100 μM) as indicated. Plates were incubated for 48 h at 28°C.
(TIF)

S9 Fig Analysis of mCherry fusion protein expression. Expression of indicated fusion proteins was analyzed by Western hybridization in derivatives of *U*. *maydis* strain SG200Δ*spp1*. Proteins were expressed under the control of the constitutive active *otef* promoter. Protein extracts were prepared from exponentially growing cells cultured in CMG liquid medium. Ponceau S-stained membranes were used as loading control. No signal was detected for SppA-mC (*A*. *nidulans*) and Ypf1-mC (*S*. *cerevisiae*).
(TIF)

S10 Fig Deletion of the *A. fumigatus srbA* ortholog *srb1* in *U.* maydis does not affect ER stress resistance or virulence. **(A)** *U*. *maydis* strain SG200 (WT) and the Δ*srb1* derivative were inoculated into 7 day-old maize seedlings. Disease symptoms were rated 8 dpi and grouped into categories depicted on the right. n represents the total number of inoculated plants from three independent experiments. **(B)** ER stress assay of *U*. *maydis* strain SG200 (WT) and the Δ*srb1* derivative. Serial 10-fold dilutions were spotted on YNBG solid medium supplemented with TM (0.5 μg/mL) as indicated. Plates were incubated for 48 h at 28°C.
(TIF)

S11 Fig Deletion of the heme oxygenase encoding gene *UMAG_00783* does not affect virulence of *U maydis*. **(A)** *U*. *maydis* strain SG200 (WT) and the Δ*UMAG_00783* derivative were inoculated into 7 day-old maize seedlings. Disease symptoms were rated 8 dpi and grouped into categories depicted on the right. n represents the total number of inoculated plants from three independent experiments.
(TIF)

S12 Fig Secretion assay of Pit2-mC in WT, Δ*cib1* and Δ*spp1* derivatives. **(A)** Secretion of Pit2-mCherry was investigated by Western hybridization of protein extracts prepared from indicated strains expressing the Pit2-mCherry fusion protein under the control of the constitutive *otef* promoter. Strains were grown in CMG with or without 5 μg/ml TM (+) and were further incubated for 4 h at 28°C. Cell pellets and supernatant were separated by centrifugation. Proteins were separated by SDS-PAGE analysis followed by Western hybridization using an mCherry specific antibody.
(TIF)

S13 Fig Secretion assay of Pep1-mC, Tin2-mC and Cmu1-mC in WT and the Δ*spp1* derivative. **(A)** Secretion of Pep1-mC, Tin2-mC and Cmu1-mC was investigated by Western hybridization of protein extracts prepared from indicated strains expressing the respective mCherry fusion proteins under the control of the constitutive *otef* promoter. Strains were grown in CMG with or without 5 μg/ml TM (+) and were further incubated for 4 h at 28°C. Cell pellets and supernatant were separated by centrifugation. Proteins were separated by SDS-PAGE analysis followed by Western hybridization using an mCherry specific antibody.
(TIF)

S14 Fig Δ*spp1* strains do not show increased expression of fungal UPR marker genes *in planta*. qRT-PCR analysis was used to monitor fungal UPR gene expression *in planta*. Indicated *U*. *maydis* strains were inoculated in 7 day-old maize seedlings and infected leaf material was collected at 2 dpi. Expression levels are depicted relative to WT infected plants and represent the mean of three biological replicates with two technical duplicates each. *eIF2b* was used for normalization. Error bars represent the SD.
(TIF)

S15 Fig Δspp1 strains are not impaired in H2O2 resistance. **(A)** H_2_O_2_ resistance of *U*. *maydis* strain SG200 (WT) and the Δ*spp1* derivative was tested by serial 10-fold dilutions of strains, spotted on YNBG solid medium supplemented with the indicated concentration of H_2_O_2_. Plates were incubated for 48 h at 28°C. **(B)** *U*. *maydis* strain SG200 (WT) and the Δ*spp1* derivative were inoculated into 7 day-old maize seedlings. Cultures used for infection experiments were supplemented with 0.5 μM (f.c.) DPI or an equivalent volume of solvent (DMSO). Disease symptoms were rated 8 dpi and grouped into categories depicted on the right. n represents the total number of inoculated plants from three independent experiments. **(C)** Fungal morphology of SG200 (WT) and the Δ*spp1* was investigated by Chlorazol Black E staining of DPI or mock (DMSO) treated infected leaf samples at 3 dpi. WT strains showed extensive proliferation *in planta*, whereas the Δ*spp1* mutant showed strongly reduced proliferation after plant penetration that was not rescued by DPI treatment. Scale bar = 20 μm.
(TIF)

S16 Fig Analysis of PR3 and PR4 gene expression in infected maize tissue. qRT-PCR based expression analysis of SA marker genes *PR3* and *PR4* in response to infection with indicated *U*. *maydis* strains. 7 day-old maize seedlings were used for inoculation and samples of infected leaf tissue were collected 2 dpi. Expression levels are depicted relative to plants infected with the WT and represent the mean of three biological replicates with two technical duplicates each. GAPDH was used for normalization. Error bars represent the SD. Statistical significance was calculated using Student’s *t* test. *P value ≤ 0.05 and ***P value ≤ 0.001.
(TIF)

S17 Fig Analysis of UPREs in the promoter region of *spp1* in related smut species. The identified Cib1 binding motif of the WT strain was subjected to the MAST (Motif Alignment & Search Tool, http://meme-suite.org/tools/mast) for motif search in the *SPP* promoter region of *U*. *maydis*, *S*. *reilianum*, *U*. *hordei* and *U*. *bromivora*. **(A)** Schematic representation of identified UPREs (red boxes). Promoter regions and genes are highlighted in grey and blue, respectively. Transcription start sites (tss) are indicated by arrows. **(B)** List of identified UPREs in consecutive order. Nucleotides of UPREs are highlighted in their respective color. P value represents the probability of a single random subsequence of the length of the motif scoring at least as good as the observed match. The identified Cib1 binding motif of the WT described in Fig 3A, is depicted in both orientations.
(TIF)

S1 Table Normalized expression of *U. maydis* genes (RPKM) and comparison between strains. (XLSX)

S2 Table FunCat analysis of UPR core genes. (XLSX)

S3 Table UPR core gene expression after TM treatment and during biotrophic development. (XLSX)

S4 Table Peaks identified by ChIP seq analysis in strains JB1*cib1-3xHA* and UVO151*cib1-3xHA*. (XLSX)

S5 Table Strains used in this study. (DOCX)

S6 Table Primers used in this study. (XLSX)
