# Supplementary figures and images for: Signal peptide peptidase activity connects the unfolded protein response to plant defense suppression by Ustilago maydis
Source: PLoS Pathog. 2019 Apr 18;15(4):e1007734. doi: 10.1371/journal.ppat.1007734 (PMC6490947; doi:10.1371/journal.ppat.1007734)

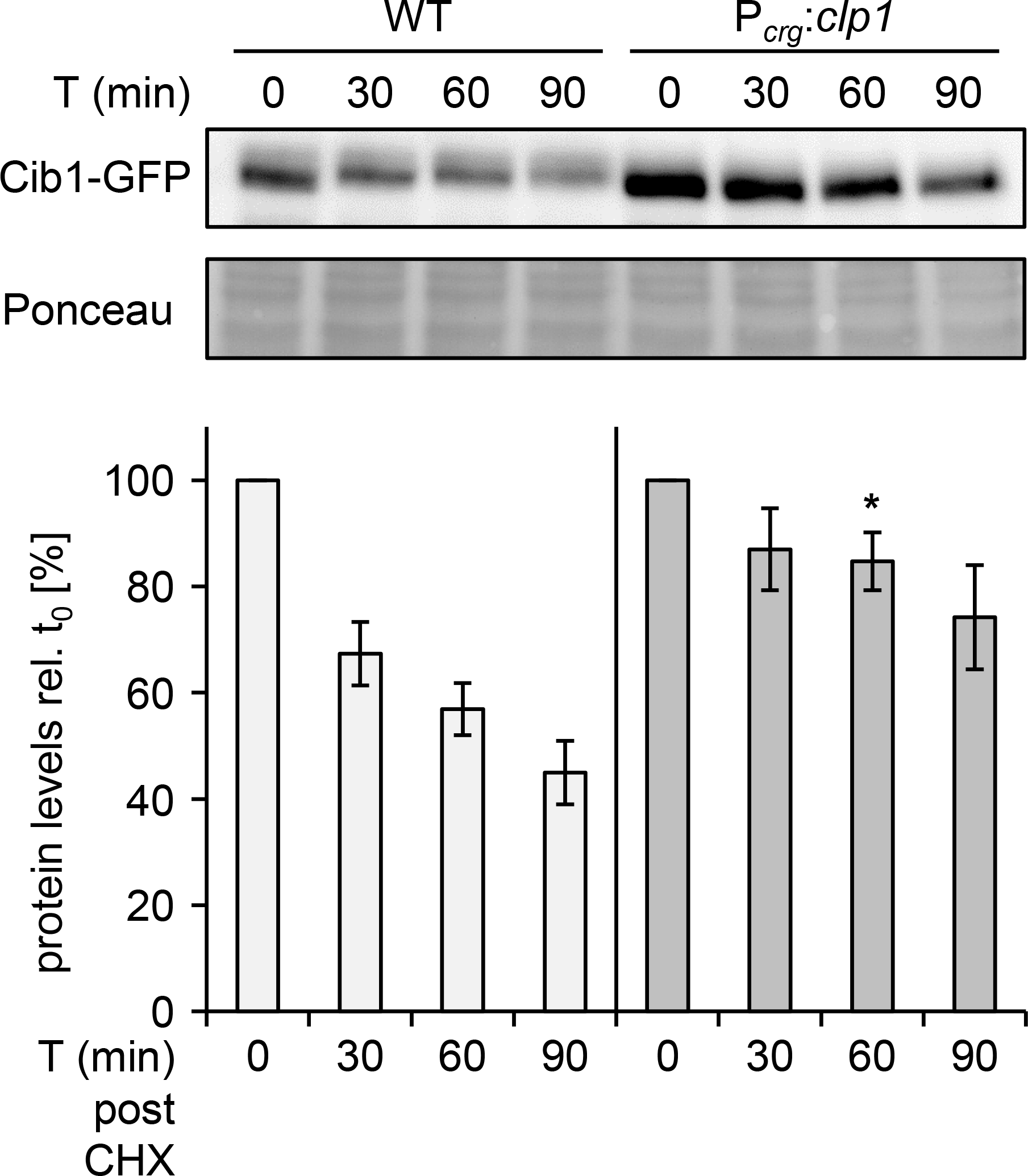

Supplement: S1 Fig — Exponentially growing strains JB1cib1-GFP (WT) and UVO151-cib1-GFP (Pcrg:clp1) were shifted to CMA liquid medium supplemented with TM (5 μg/ml) to induce clp1 and Cib1-GFP expression, respectively. Strains were further incubated for 4 h at 28°C, and 100 μg/ml CHX was added to inhibit protein expression. Protein extracts were prepared from samples taken directly before (T0) and 30 min (T1), 60 min (T2) or 90 min (T3) after CHX treatment and analyzed by Western hybridization with GFP specific antibodies. Ponceau S-stained membranes were used as loading control and for normalization of Cib1-GFP levels. Expression levels at T1, T2 and T3 were calculated relative to T0 using ImageJ. Values represent the mean of three biological replicates and error bars indicate the SEM. Statistical significance was calculated using Student’s t test. *P value ≤ 0.05. (TIF) [file ppat.1007734.s001.tif]

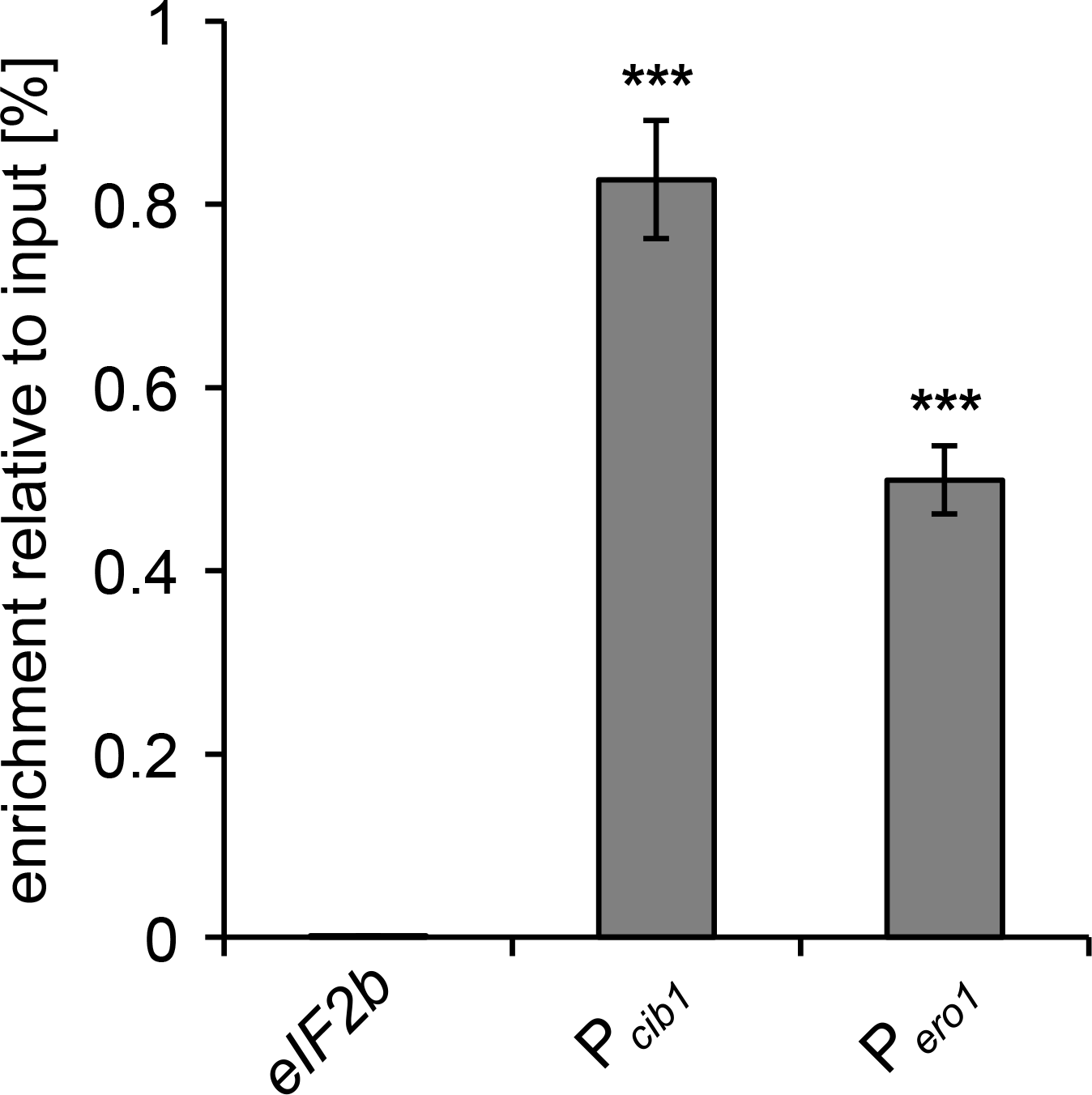

Supplement: S2 Fig — ChIP q-PCR was performed on selected promoters of the corresponding genes cib1 (UMAG_11782) and ero1 (UMAG_05219) to test for promoter enrichment. The experiment was performed as described in Fig 3A. The gene eIF2b served as negative control. Enrichment was depicted relative to input DNA. Values represent the mean of three biological replicates and two technical duplicates each. Error bars indicate the SD. Statistical significance was calculated using Student’s t test. ***P value ≤ 0.001. (TIF) [file ppat.1007734.s002.tif]

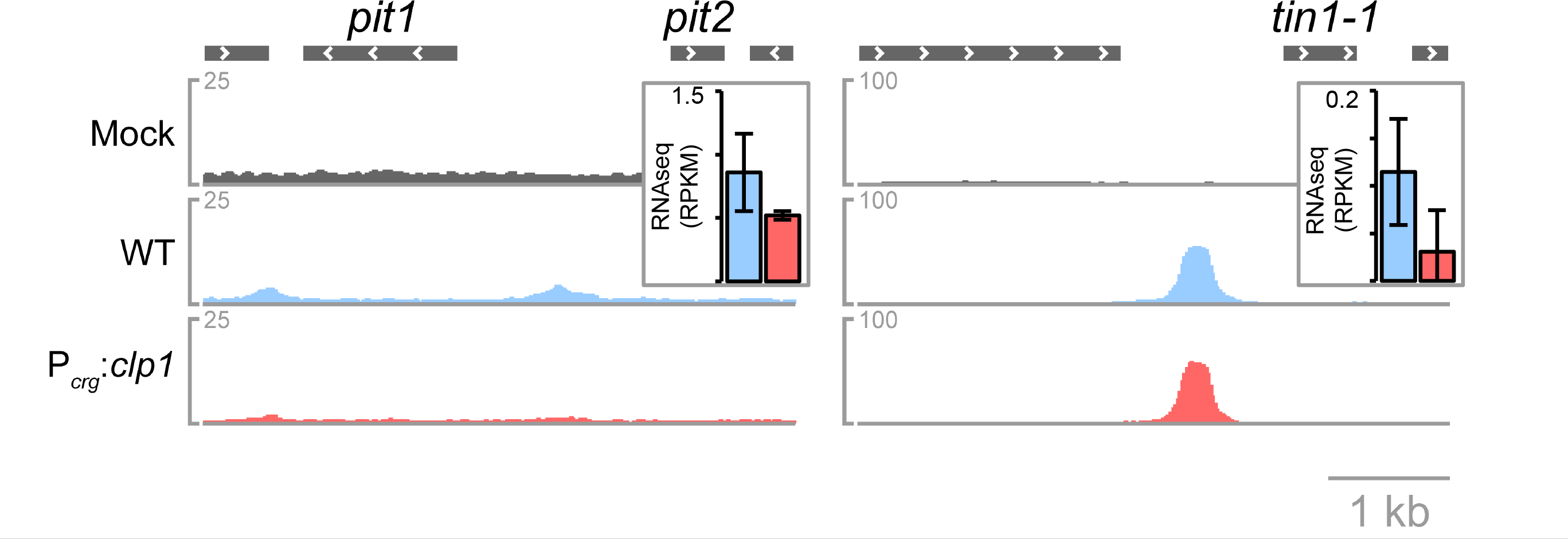

Supplement: S3 Fig — Visualization of Cib1 binding to promoters of U. maydis effector genes pit1 and tin1-1 obtained by ChIPseq analysis. Strains, growth conditions and visualization of data was performed as described in Fig 3A. (TIF) [file ppat.1007734.s003.tif]

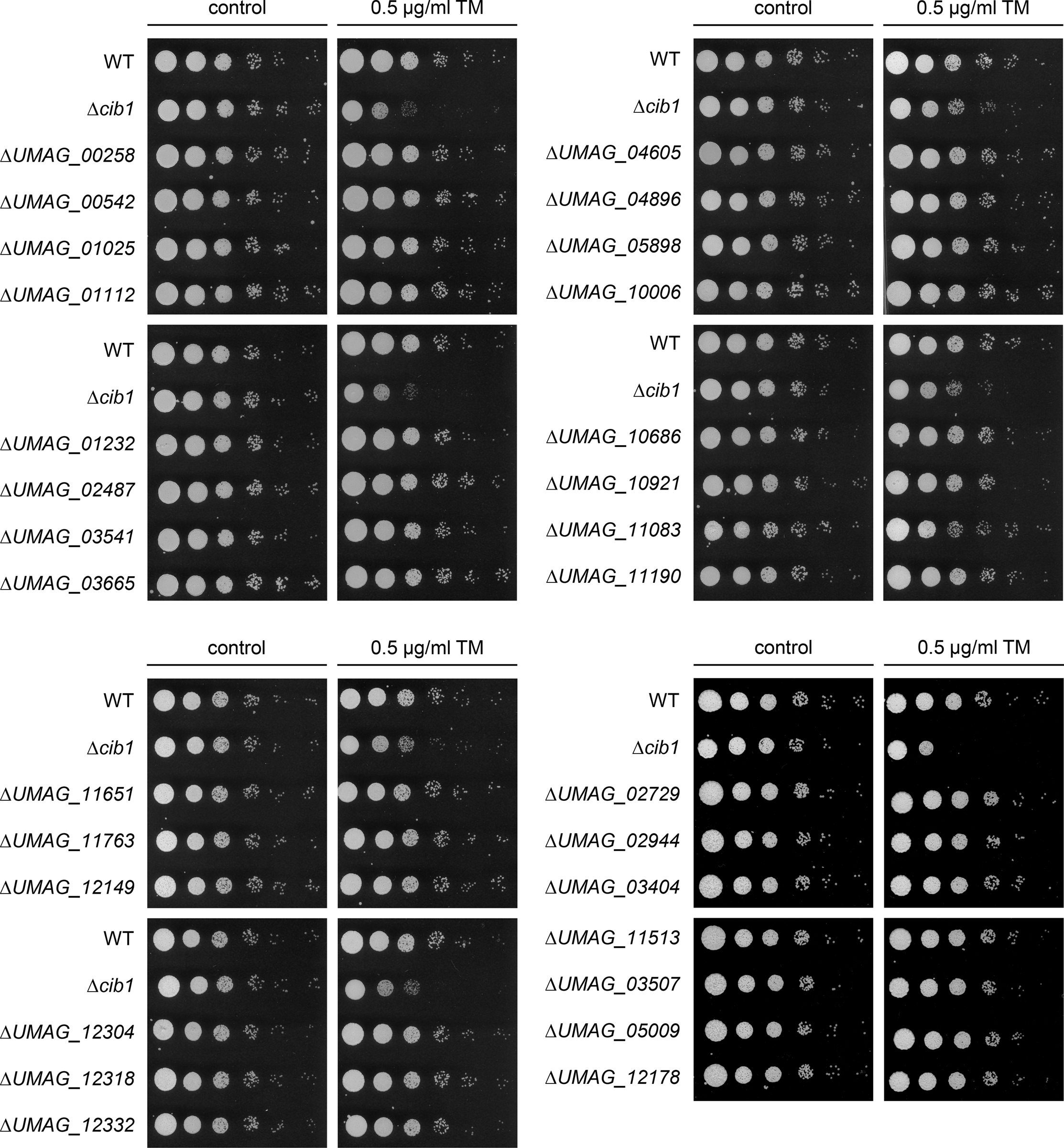

Supplement: S4 Fig — ER stress assay of U. maydis strain SG200 (WT) and derivatives. Serial 10-fold dilutions were spotted on YNBG solid medium supplemented with TM (0.5 μg/ml) as indicated. Plates were incubated for 48 h at 28°C. (TIF) [file ppat.1007734.s004.tif]

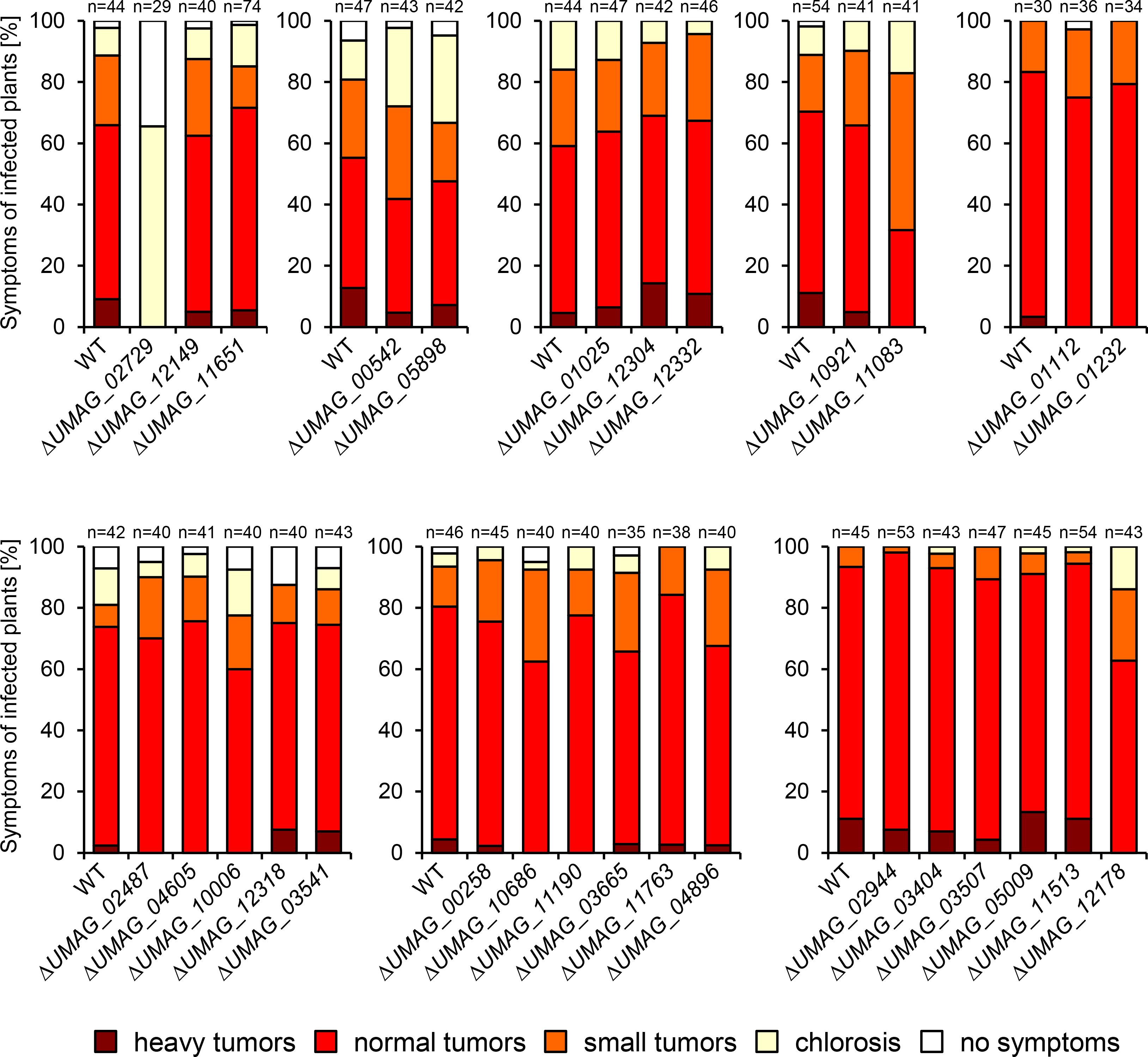

Supplement: S5 Fig — U. maydis strain SG200 (WT) and derivatives were inoculated into 7 day-old maize seedlings. Disease symptoms were rated 8 d after inoculation and grouped into categories depicted below. n represents the number of inoculated plants in a single infection experiment. (TIF) [file ppat.1007734.s005.tif]

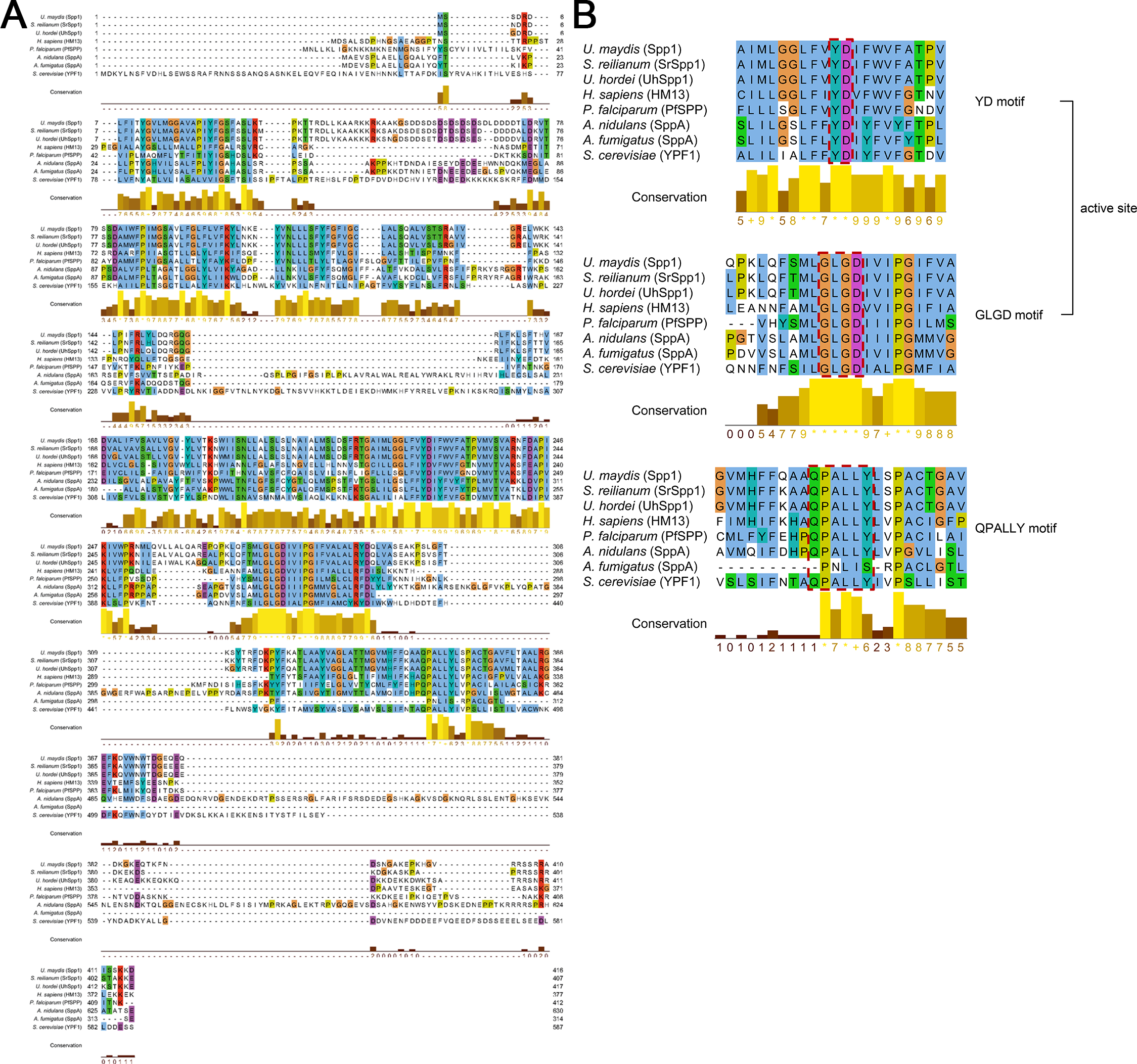

Supplement: S6 Fig — Protein sequences of U. maydis Spp1 and predicted orthologs from indicated species were aligned using the MUSCLE algorithm (https://www.ebi.ac.uk/Tools/msa/muscle) and visualized by JalView (http://www.jalview.org). Full alignment is shown in (A), and conserved sequence motifs are highlighted in (B). The YD and GLGD motifs represent the active site of the aligned signal peptide peptidases. The QPALLY motif is conserved in all sequences except for Aspergillus fumigatus. Conservation of the sequence is shown on base of physico-chemical properties in the histogram below. + indicates that all properties are conserved (10) and values can go up to 11 (marked by *) in case of full conservation and identical amino acids. The Clustal X color scheme was used to group amino acids with similar properties. (TIF) [file ppat.1007734.s006.tif]

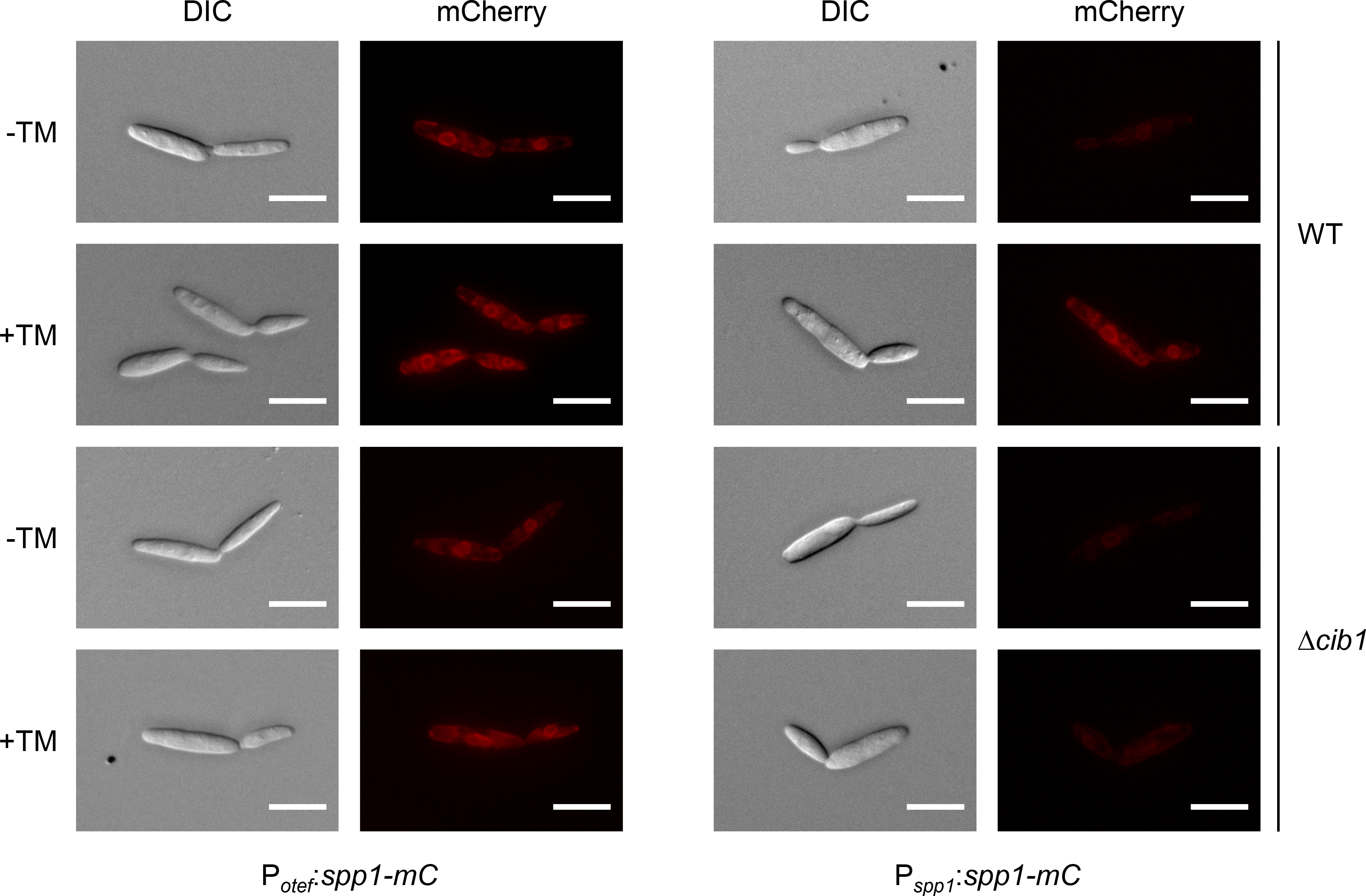

Supplement: S7 Fig — Localization and accumulation of the Spp1-mCherry fusion protein was monitored by fluorescence microscopy. Spp1-mC was expressed under the control of the native spp1- or the constitutive otef-promoter in WT and Δcib1 mutant background. Cells were analyzed 2 h after TM-mediated UPR induction and compared to the untreated control. Cellular morphology was visualized by DIC microscopy. Scale bars = 10 μm. (TIF) [file ppat.1007734.s007.tif]

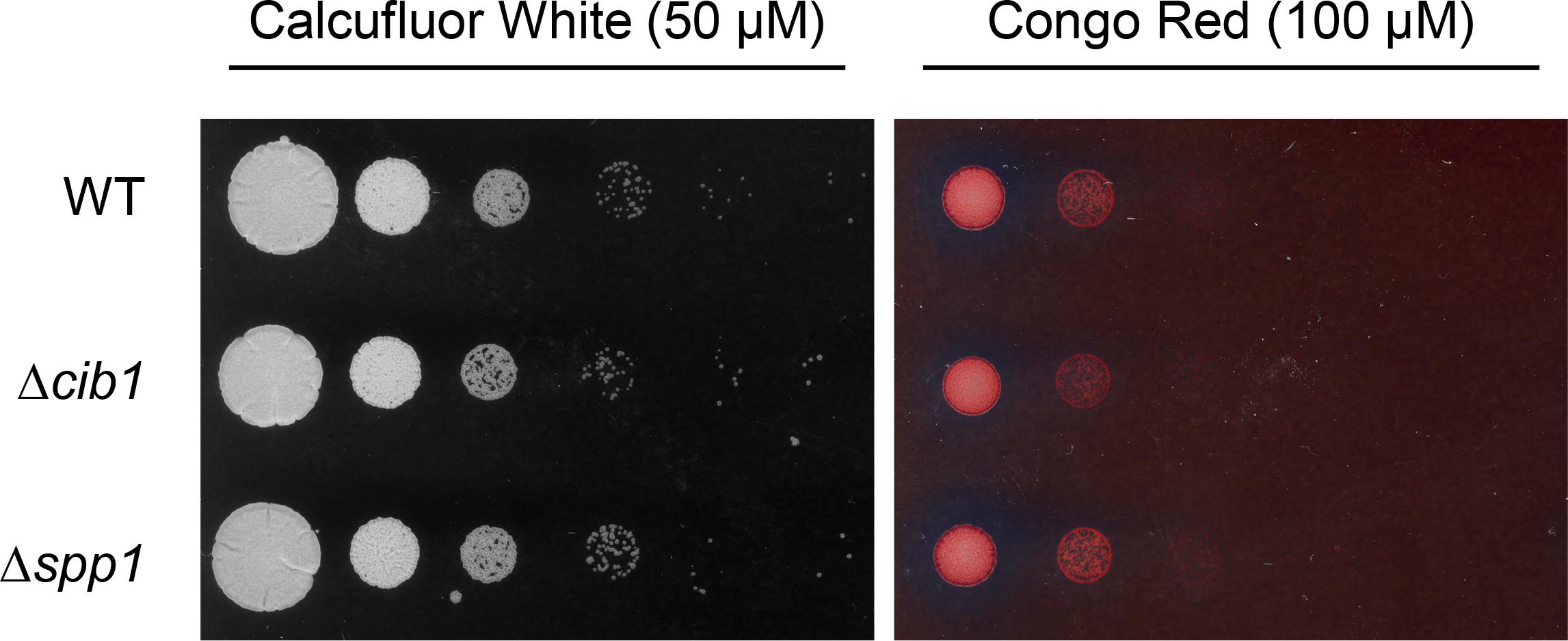

Supplement: S8 Fig — Cell wall stress resistance of U. maydis strain SG200 (WT) and the Δspp1 derivative was tested by serial 10-fold dilutions of strains, spotted on YNBG solid medium supplemented with Calcofluor White (50 μM) or Congo Red (100 μM) as indicated. Plates were incubated for 48 h at 28°C. (TIF) [file ppat.1007734.s008.tif]

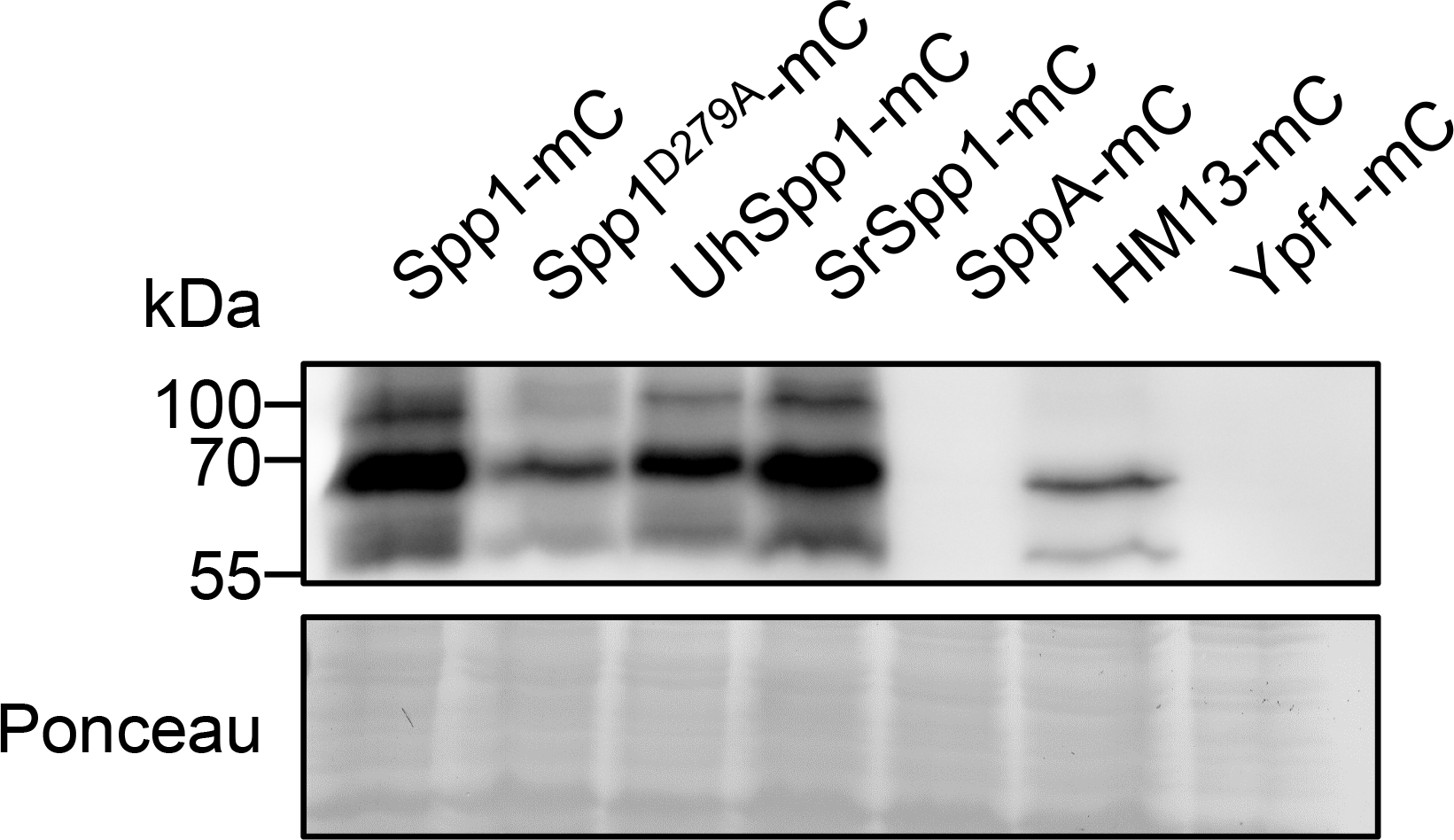

Supplement: S9 Fig — Expression of indicated fusion proteins was analyzed by Western hybridization in derivatives of U. maydis strain SG200Δspp1. Proteins were expressed under the control of the constitutive active otef promoter. Protein extracts were prepared from exponentially growing cells cultured in CMG liquid medium. Ponceau S-stained membranes were used as loading control. No signal was detected for SppA-mC (A. nidulans) and Ypf1-mC (S. cerevisiae). (TIF) [file ppat.1007734.s009.tif]

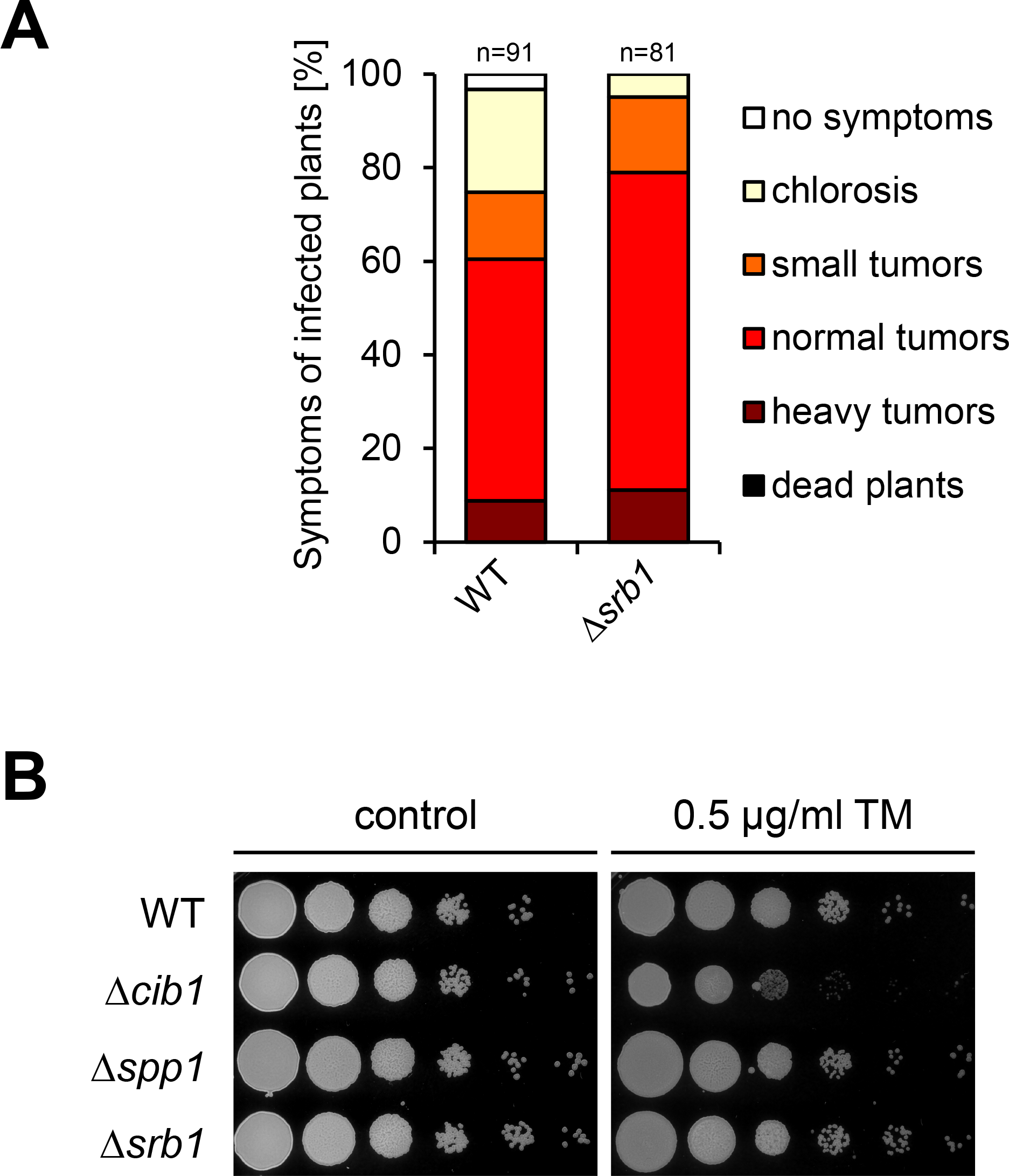

Supplement: S10 Fig — (A) U. maydis strain SG200 (WT) and the Δsrb1 derivative were inoculated into 7 day-old maize seedlings. Disease symptoms were rated 8 dpi and grouped into categories depicted on the right. n represents the total number of inoculated plants from three independent experiments. (B) ER stress assay of U. maydis strain SG200 (WT) and the Δsrb1 derivative. Serial 10-fold dilutions were spotted on YNBG solid medium supplemented with TM (0.5 μg/mL) as indicated. Plates were incubated for 48 h at 28°C. (TIF) [file ppat.1007734.s010.tif]

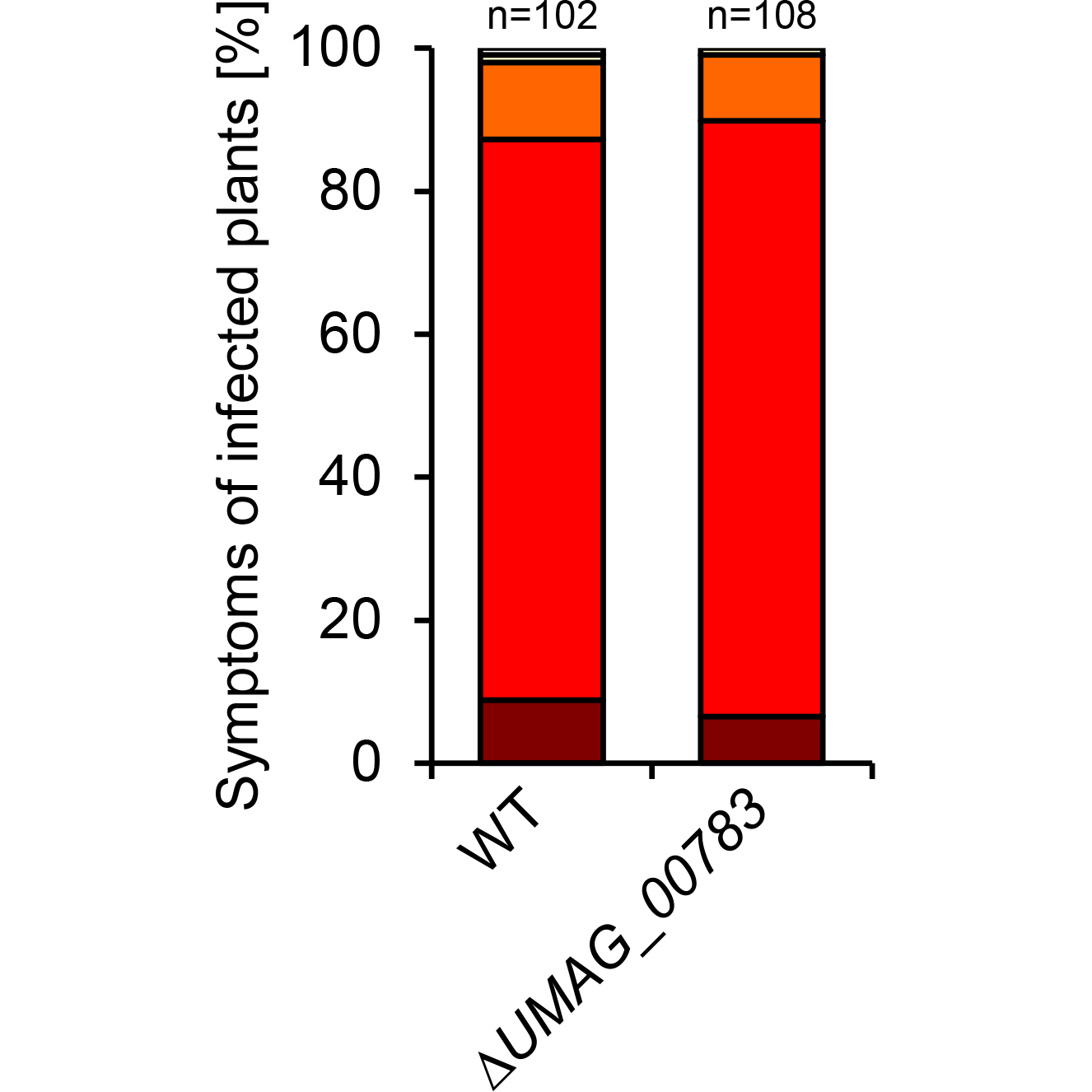

Supplement: S11 Fig — (A) U. maydis strain SG200 (WT) and the ΔUMAG_00783 derivative were inoculated into 7 day-old maize seedlings. Disease symptoms were rated 8 dpi and grouped into categories depicted on the right. n represents the total number of inoculated plants from three independent experiments. (TIF) [file ppat.1007734.s011.tif]

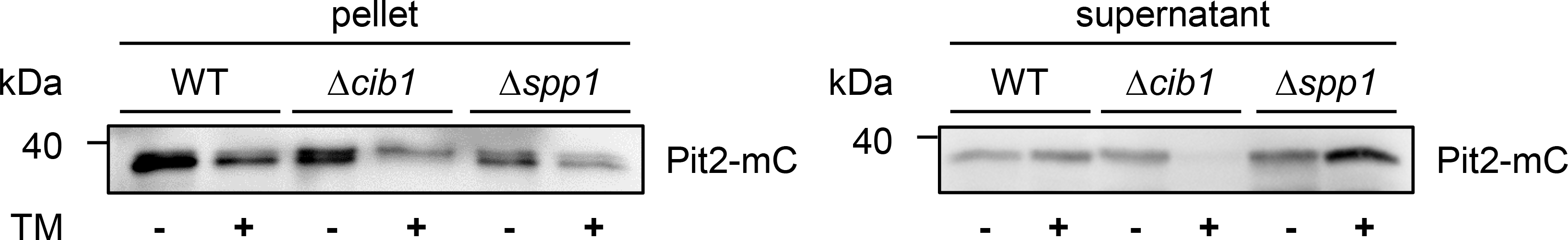

Supplement: S12 Fig — (A) Secretion of Pit2-mCherry was investigated by Western hybridization of protein extracts prepared from indicated strains expressing the Pit2-mCherry fusion protein under the control of the constitutive otef promoter. Strains were grown in CMG with or without 5 μg/ml TM (+) and were further incubated for 4 h at 28°C. Cell pellets and supernatant were separated by centrifugation. Proteins were separated by SDS-PAGE analysis followed by Western hybridization using an mCherry specific antibody. (TIF) [file ppat.1007734.s012.tif]

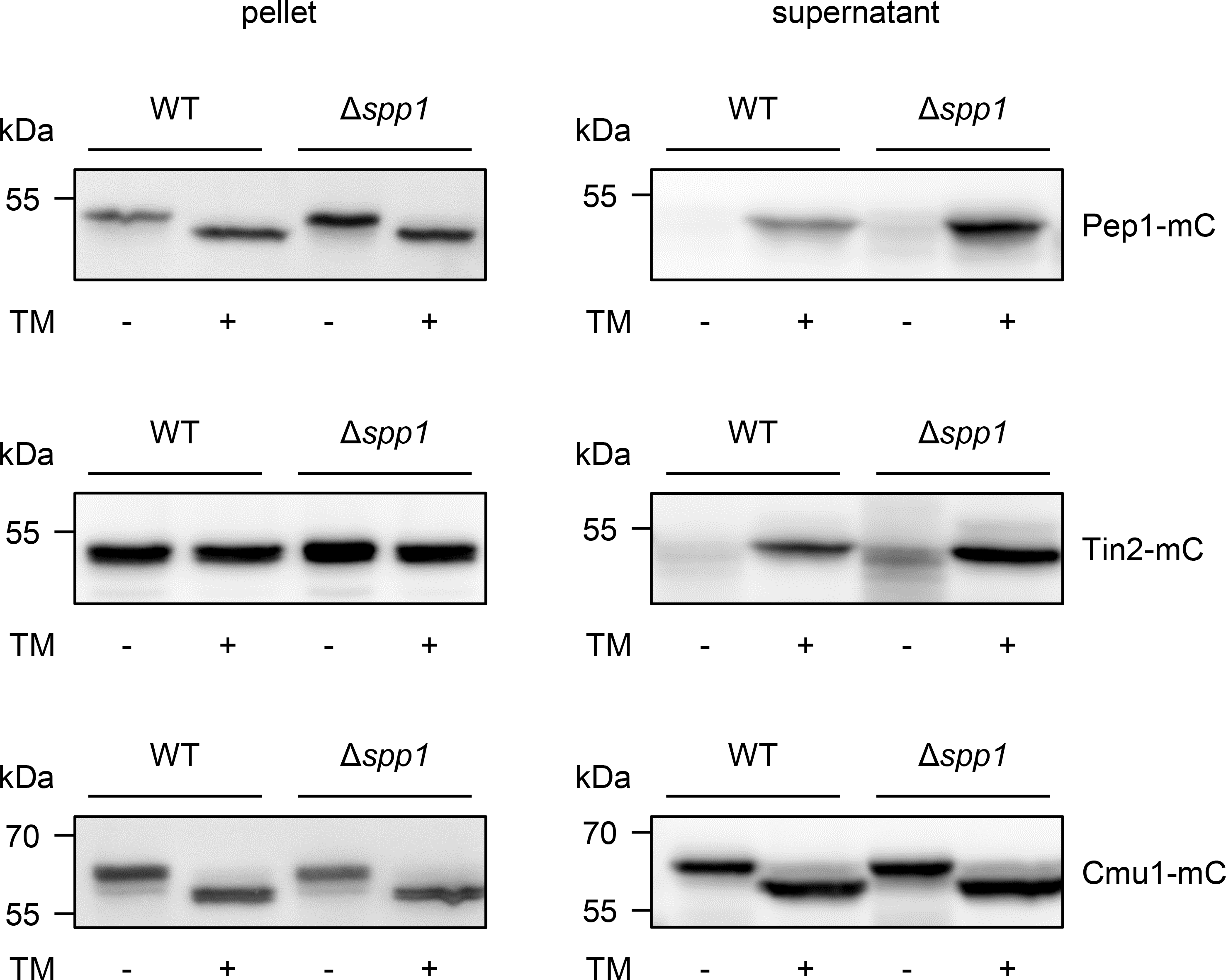

Supplement: S13 Fig — (A) Secretion of Pep1-mC, Tin2-mC and Cmu1-mC was investigated by Western hybridization of protein extracts prepared from indicated strains expressing the respective mCherry fusion proteins under the control of the constitutive otef promoter. Strains were grown in CMG with or without 5 μg/ml TM (+) and were further incubated for 4 h at 28°C. Cell pellets and supernatant were separated by centrifugation. Proteins were separated by SDS-PAGE analysis followed by Western hybridization using an mCherry specific antibody. (TIF) [file ppat.1007734.s013.tif]

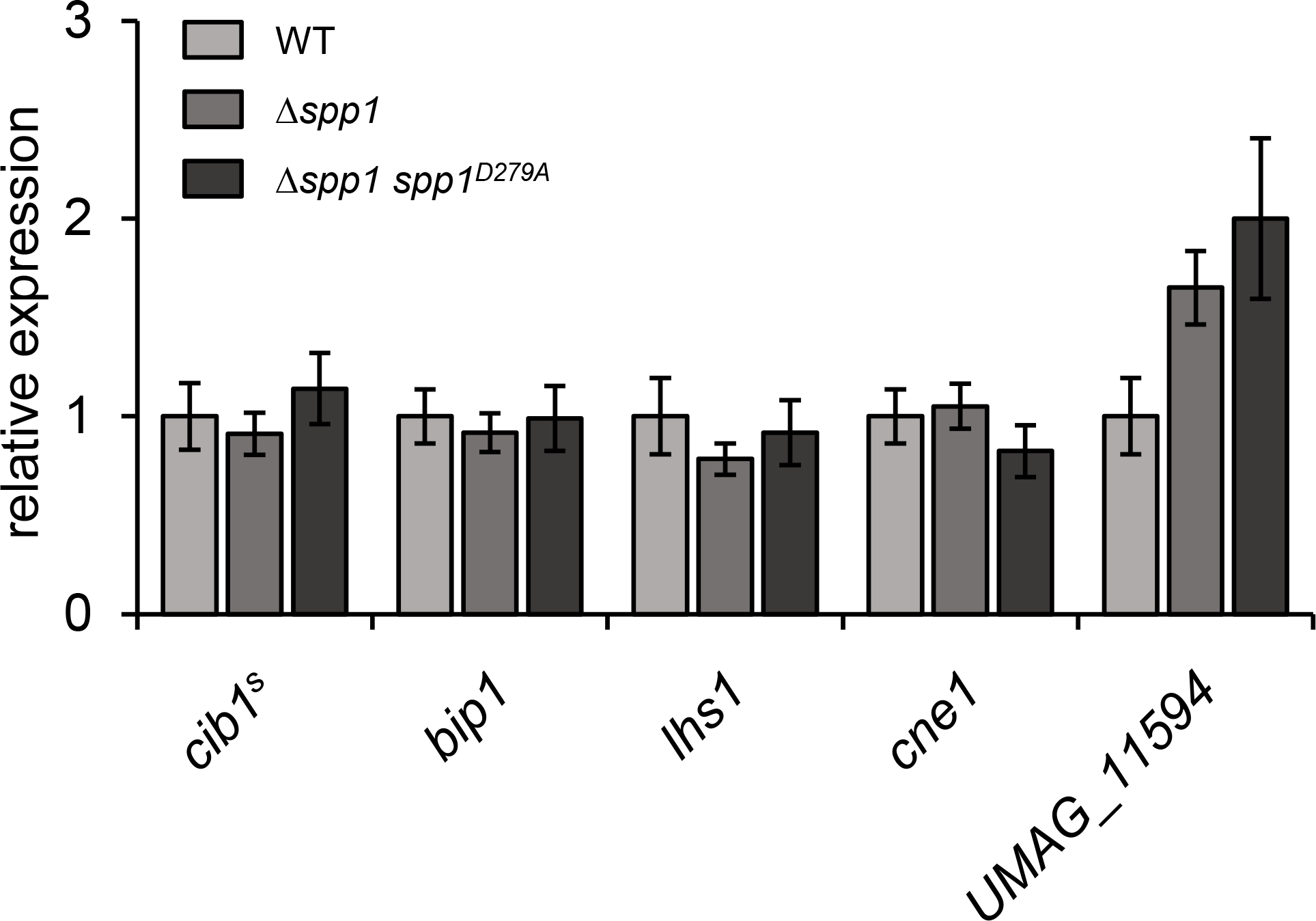

Supplement: S14 Fig — qRT-PCR analysis was used to monitor fungal UPR gene expression in planta. Indicated U. maydis strains were inoculated in 7 day-old maize seedlings and infected leaf material was collected at 2 dpi. Expression levels are depicted relative to WT infected plants and represent the mean of three biological replicates with two technical duplicates each. eIF2b was used for normalization. Error bars represent the SD. (TIF) [file ppat.1007734.s014.tif]

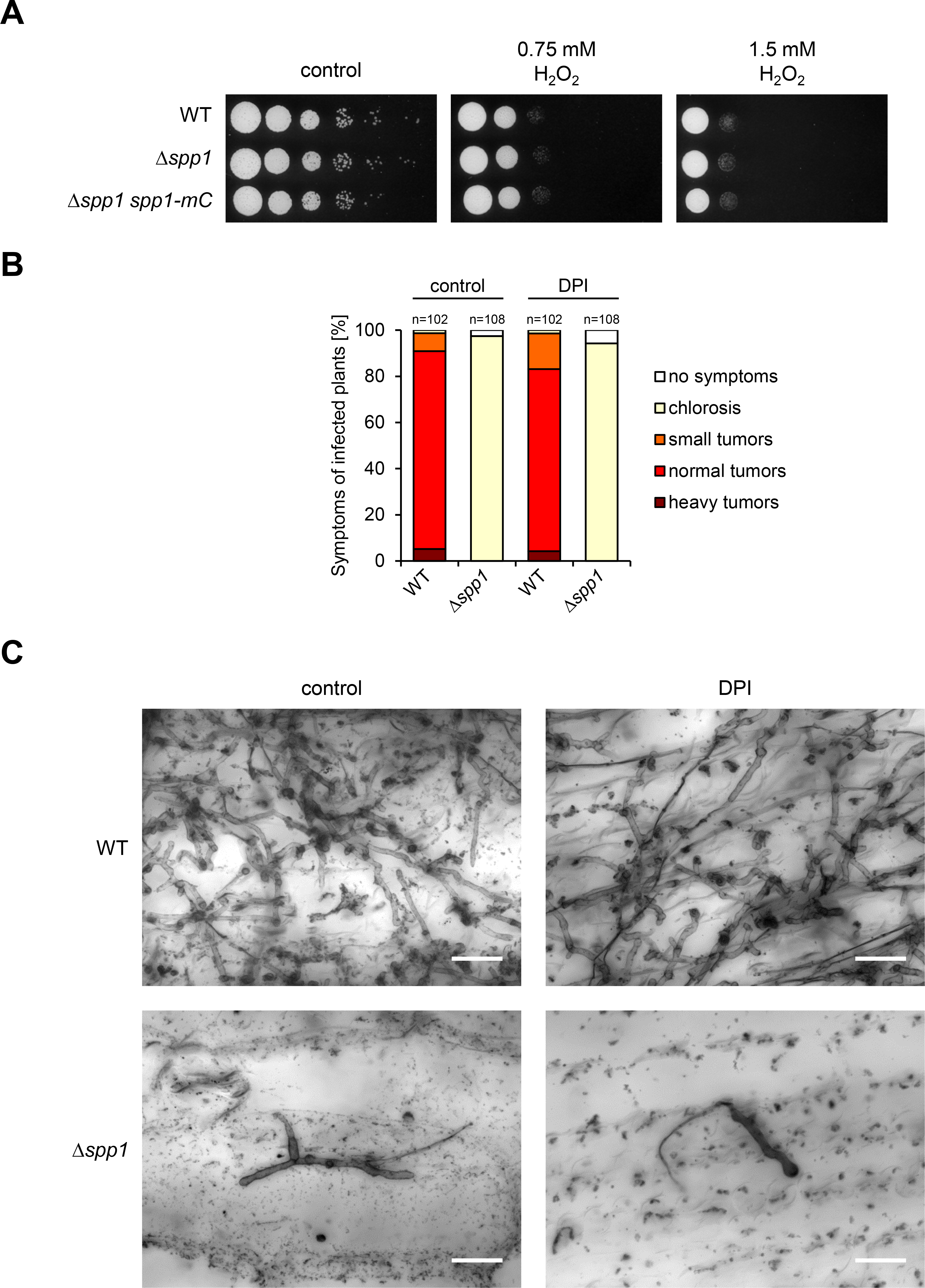

Supplement: S15 Fig — (A) H2O2 resistance of U. maydis strain SG200 (WT) and the Δspp1 derivative was tested by serial 10-fold dilutions of strains, spotted on YNBG solid medium supplemented with the indicated concentration of H2O2. Plates were incubated for 48 h at 28°C. (B) U. maydis strain SG200 (WT) and the Δspp1 derivative were inoculated into 7 day-old maize seedlings. Cultures used for infection experiments were supplemented with 0.5 μM (f.c.) DPI or an equivalent volume of solvent (DMSO). Disease symptoms were rated 8 dpi and grouped into categories depicted on the right. n represents the total number of inoculated plants from three independent experiments. (C) Fungal morphology of SG200 (WT) and the Δspp1 was investigated by Chlorazol Black E staining of DPI or mock (DMSO) treated infected leaf samples at 3 dpi. WT strains showed extensive proliferation in planta, whereas the Δspp1 mutant showed strongly reduced proliferation after plant penetration that was not rescued by DPI treatment. Scale bar = 20 μm. (TIF) [file ppat.1007734.s015.tif]

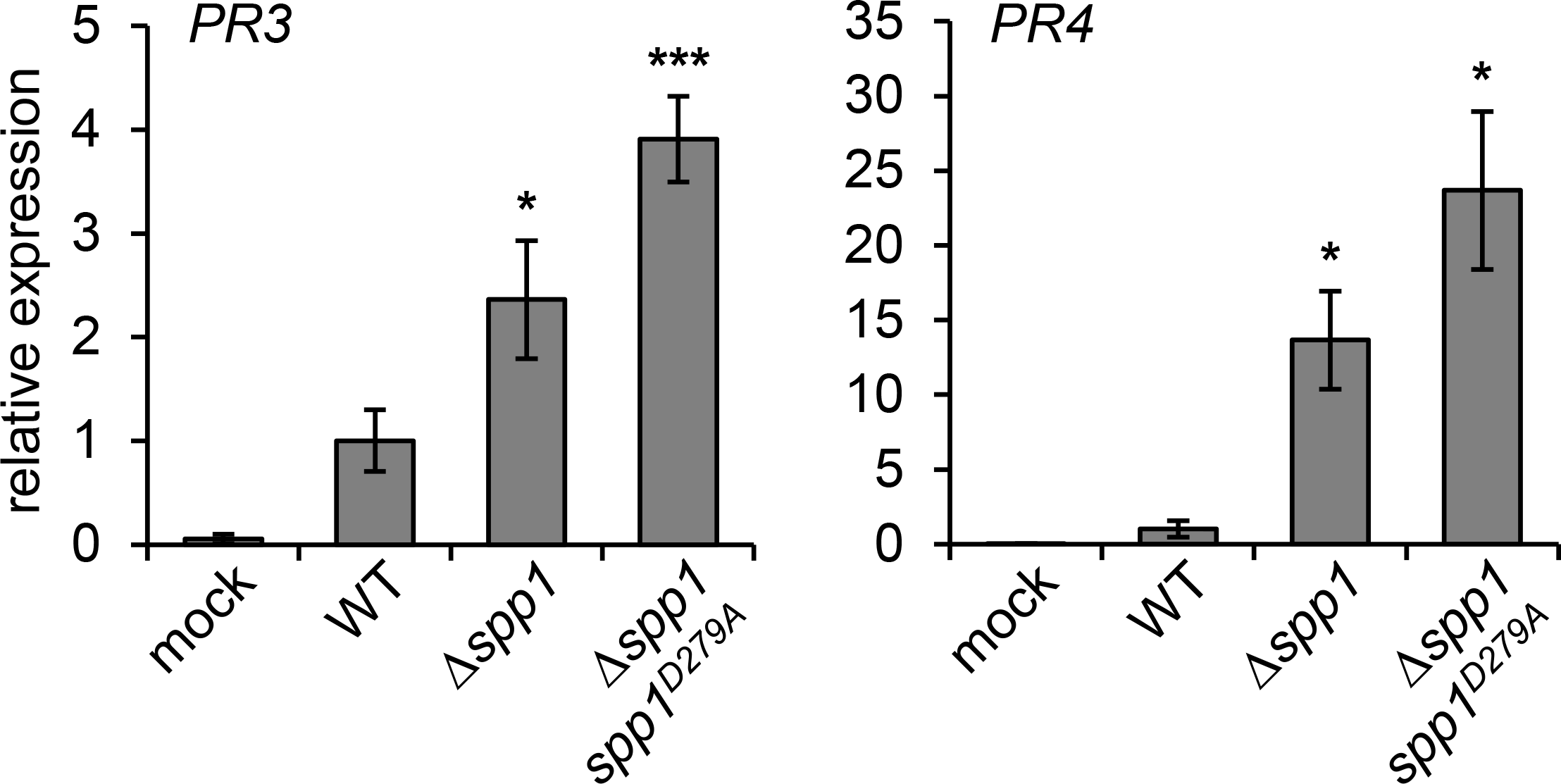

Supplement: S16 Fig — qRT-PCR based expression analysis of SA marker genes PR3 and PR4 in response to infection with indicated U. maydis strains. 7 day-old maize seedlings were used for inoculation and samples of infected leaf tissue were collected 2 dpi. Expression levels are depicted relative to plants infected with the WT and represent the mean of three biological replicates with two technical duplicates each. GAPDH was used for normalization. Error bars represent the SD. Statistical significance was calculated using Student’s t test. *P value ≤ 0.05 and ***P value ≤ 0.001. (TIF) [file ppat.1007734.s016.tif]

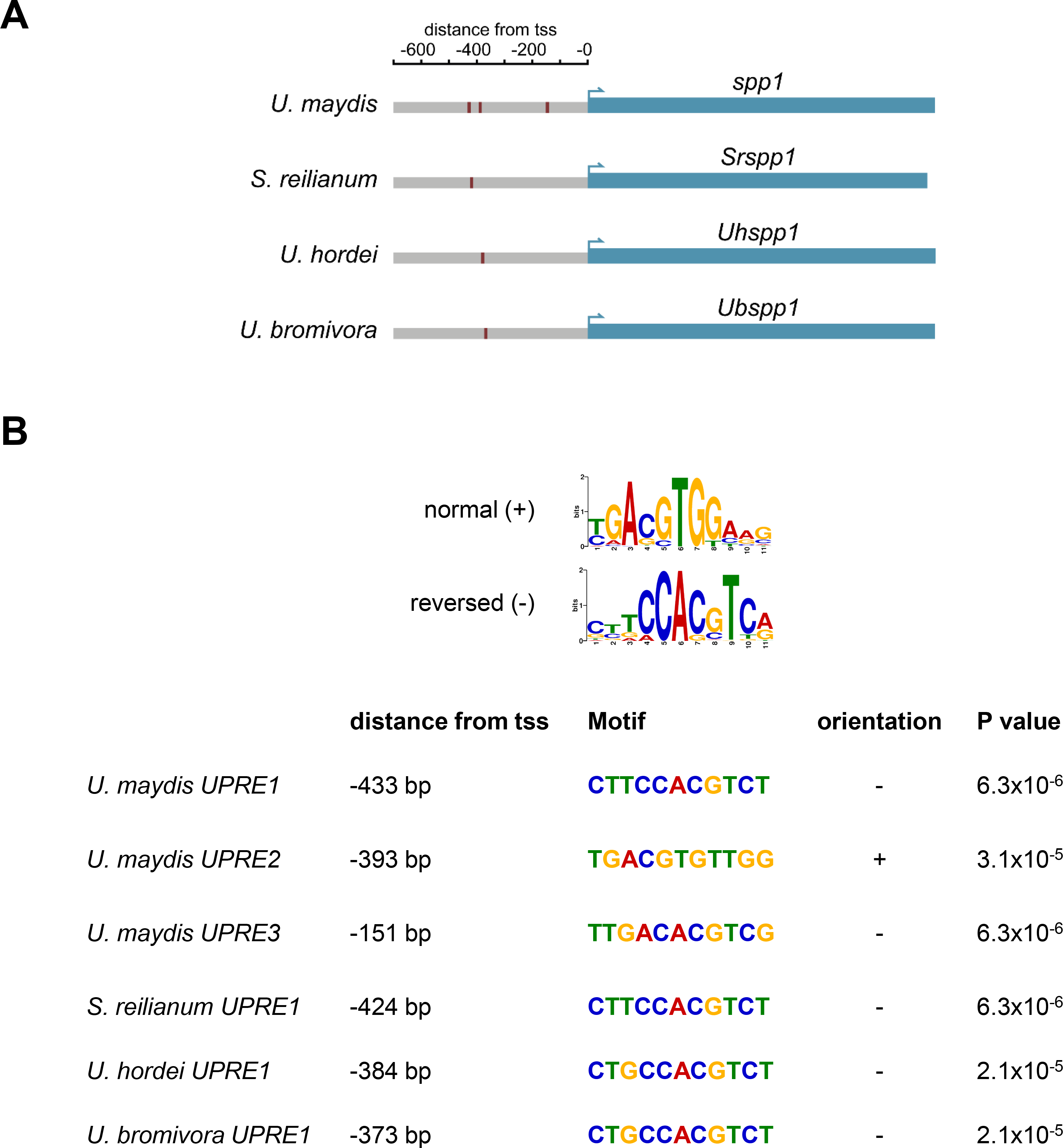

Supplement: S17 Fig — The identified Cib1 binding motif of the WT strain was subjected to the MAST (Motif Alignment & Search Tool, http://meme-suite.org/tools/mast) for motif search in the SPP promoter region of U. maydis, S. reilianum, U. hordei and U. bromivora. (A) Schematic representation of identified UPREs (red boxes). Promoter regions and genes are highlighted in grey and blue, respectively. Transcription start sites (tss) are indicated by arrows. (B) List of identified UPREs in consecutive order. Nucleotides of UPREs are highlighted in their respective color. P value represents the probability of a single random subsequence of the length of the motif scoring at least as good as the observed match. The identified Cib1 binding motif of the WT described in Fig 3A, is depicted in both orientations. (TIF) [file ppat.1007734.s017.tif]
