# Supplementary material for: Signal peptide peptidase activity connects the unfolded protein response to plant defense suppression by Ustilago maydis
Source: PLoS Pathog. 2019 Apr 18;15(4):e1007734. doi: 10.1371/journal.ppat.1007734 (PMC6490947; doi:10.1371/journal.ppat.1007734)
Supplement: S5 Table — (DOCX) [file ppat.1007734.s022.docx]

**S4 Table: Strains used in this study**

| **Strain** | **Genotype** | **Reference** |
| --- | --- | --- |
| JB1 | *a1* ∆*b* | Scherer *et al.*, 2006^(1)^ |
| JB1∆*cib1* | *a1* ∆*b* ∆*cib1* | Heimel *et al.*, 2013^(2)^ |
| JB1*cib1-GFP* | *a1* ∆*b cib1-GFP* | this study |
| JB1*cib1-3xHA* | *a1* ∆*b cib1-3xHA* | this study |
| JB1*P_tetO_:cib1-GFP* | *a1* ∆*b P_tef_:tTA* P_tetO_:cib1-GFP* | this study |
| UVO151 | *a1* ∆*b P_crg1_:clp1* | Scherer *et al.*, 2006^(1)^ |
| UVO151*cib1-GFP* | *a1* ∆*b P_crg1_:clp1 cib1-GFP* | this study |
| UVO151*cib1-3xHA* | *a1* ∆*b P_crg1_:clp1 cib1-3xHA* | this study |
| UVO151*P_tetO_:cib1-GFP* | *a1* ∆*b P_crg1_:clp1 P_tef_:tTA* P_tetO_:cib1-GFP* | this study |
| SG200 | *a1:mfa2 bE1 bW2* | Kämper *et al.*, 2006^(3)^ |
| SG200∆*cib1* | *a1:mfa2 bE1 bW2* ∆*cib1* | Heimel *et al.*, 2010^(4)^ |
| SG200∆*hrd1* | *a1:mfa2 bE1 bW2* ∆*UMAG_00542* | this study |
| SG200∆*UMAG_00783* | *a1:mfa2 bE1 bW2* ∆*UMAG_00783* | this study |
| SG200∆*UMAG_01025* | *a1:mfa2 bE1 bW2* ∆*UMAG_01025* | this study |
| SG200∆*UMAG_01112* | *a1:mfa2 bE1 bW2* ∆*UMAG_01112* | this study |
| SG200∆*UMAG_01232* | *a1:mfa2 bE1 bW2* ∆*UMAG_01232* | this study |
| SG200∆*UMAG_02487* | *a1:mfa2 bE1 bW2* ∆*UMAG_02487* | this study |
| SG200∆*spp1* | *a1:mfa2 bE1 bW2* ∆*UMAG_02729* | this study |
| SG200∆*UMAG_02944* | *a1:mfa2 bE1 bW2* ∆*UMAG_02944* | this study |
| SG200∆*UMAG_03404* | *a1:mfa2 bE1 bW2* ∆*UMAG_03404* | this study |
| SG200∆*UMAG_03507* | *a1:mfa2 bE1 bW2* ∆*UMAG_03507* | this study |
| SG200∆*UMAG_03541* | *a1:mfa2 bE1 bW2* ∆*UMAG_03541* | this study |
| SG200∆*UMAG_03665* | *a1:mfa2 bE1 bW2* ∆*UMAG_03665* | this study |
| SG200∆*UMAG_04605* | *a1:mfa2 bE1 bW2* ∆*UMAG_04605* | this study |
| SG200∆*UMAG_04896* | *a1:mfa2 bE1 bW2* ∆*UMAG_04896* | this study |
| SG200∆*UMAG_05009* | *a1:mfa2 bE1 bW2* ∆*UMAG_05009* | this study |
| SG200∆*der1* | *a1:mfa2 bE1 bW2* ∆*UMAG_05898* | this study |
| SG200∆*UMAG_10006* | *a1:mfa2 bE1 bW2* ∆*UMAG_10006* | this study |
| SG200∆*UMAG_10686* | *a1:mfa2 bE1 bW2* ∆*UMAG_10686* | this study |
| SG200∆*UMAG_10921* | *a1:mfa2 bE1 bW2* ∆*UMAG_10921* | this study |
| SG200∆*UMAG_11083* | *a1:mfa2 bE1 bW2* ∆*UMAG_11083* | this study |
| SG200∆*UMAG_11190* | *a1:mfa2 bE1 bW2* ∆*UMAG_11190* | this study |
| SG200∆*UMAG_11513* | *a1:mfa2 bE1 bW2* ∆*UMAG_11513* | this study |
| SG200∆*UMAG_11651* | *a1:mfa2 bE1 bW2* ∆*UMAG_11651* | this study |
| SG200∆*UMAG_11763* | *a1:mfa2 bE1 bW2* ∆*UMAG_11763* | this study |
| SG200∆*UMAG_12149* | *a1:mfa2 bE1 bW2* ∆*UMAG_12149* | this study |
| SG200∆*UMAG_12178* | *a1:mfa2 bE1 bW2* ∆*UMAG_12178* | this study |
| SG200∆*UMAG_12304* | *a1:mfa2 bE1 bW2* ∆*UMAG_12304* | this study |
| SG200∆*UMAG_12318* | *a1:mfa2 bE1 bW2* ∆*UMAG_12318* | this study |
| SG200∆*UMAG_12332* | *a1:mfa2 bE1 bW2* ∆*UMAG_12332* | this study |
| SG200∆*doa10* | *a1:mfa2 bE1 bW2* ∆*UMAG_10911* | this study |
| SG200∆*der2* | *a1:mfa2 bE1 bW2* ∆*UMAG_11402* | this study |
| SG200∆*hrd1*∆*doa10* | *a1:mfa2 bE1 bW2* ∆*UMAG_00542* ∆*UMAG_10911* | this study |
| SG200∆*hrd1*∆*doa10*∆*der1* | *a1:mfa2 bE1 bW2* ∆*UMAG_00542* ∆*UMAG_10911* ∆*UMAG_05898* | this study |
| SG200∆*hrd1*∆*doa10*∆*der2* | *a1:mfa2 bE1 bW2* ∆*UMAG_00542* ∆*UMAG_10911* ∆*UMAG_11402* | this study |
| SG200∆*srb1* | *a1:mfa2 bE1 bW2* ∆*UMAG_05721* | this study |
| SG200∆*spp1**-P_spp1_:spp1-mCherry* | *a1:mfa2 bE1 bW2* ∆*spp1 ip^r^[P_spp1_:spp1-mCherry]ip^s^* | this study |
| SG200∆*spp1-P_otef_:spp1-mCherry* | *a1:mfa2 bE1 bW2* ∆*spp1  ip^r^[P_otef_:spp1-mCherry]ip^s^* | this study |
| SG200∆*spp1-P_spp1_:spp1-mCherry-*∆*cib1* | *a1:mfa2 bE1 bW2* ∆*spp1 ip^r^[P_spp1_:spp1-mCherry]ip^s^* ∆*cib1* | this study |
| SG200∆*spp1-P_otef_:spp1-mCherry-*∆*cib1* | *a1:mfa2 bE1 bW2* ∆*spp1  ip^r^[P_otef_:spp1-mCherry]ip^s^* ∆*cib1* | this study |
| SG200∆*spp1-P_otef_:spp1 ^(D279A)^-mCherry* | *a1:mfa2 bE1 bW2* ∆*spp1  ip^r^[P_otef_:spp1^(D279A)^-mCherry]ip^s^* | this study |
| SG200∆*spp1-P_otef_:srspp1-mCherry* | *a1:mfa2 bE1 bW2* ∆*spp1  ip^r^[P_otef_:srspp1-mCherry]ip^s^* | this study |
| SG200∆*spp1-P_otef_:uhspp1-mCherry* | *a1:mfa2 bE1 bW2* ∆*spp1  ip^r^[P_otef_:uhspp1-mCherry]ip^s^* | this study |
| SG200∆*spp1-P_otef_:HM13-mCherry* | *a1:mfa2 bE1 bW2* ∆*spp1  ip^r^[P_otef_:HM13-mCherry]ip^s^* | this study |
| SG200∆*spp1-P_otef_:HM13-mCherry* | *a1:mfa2 bE1 bW2* ∆*spp1  ip^r^[P_otef_:HM13-mCherry]ip^s^ (multiple)* | this study |
| SG200∆*spp1-P_otef_:sppA-mCherry* | *a1:mfa2 bE1 bW2* ∆*spp1  ip^r^[P_otef_:sppA-mCherry]ip^s^* | this study |
| SG200∆*spp1-P_otef_:YPF1-mCherry* | *a1:mfa2 bE1 bW2* ∆*spp1  ip^r^[P_otef_:YPF1-mCherry]ip^s^* | this study |
| SG200∆pit2-P_otef_:pit2-mCherry | *a1:mfa2 bE1 bW2 ∆pit2 ip^r^[P_otef_:pit2-mCherry]ip^s^* | Hampel *et al.*, 2016^(5)^ |
| SG200∆pit2-P_otef_:pit2-mCherry*-*∆*cib1* | *a1:mfa2 bE1 bW2* ∆*pit2  ip^r^[P_otef_:pit2-mCherry]ip^s^* ∆*cib1* | Hampel *et al.*, 2016^(5)^ |
| SG200∆pit2-P_otef_:pit2-mCherry*-*∆*spp1* | *a1:mfa2 bE1 bW2* ∆*pit2  ip^r^[P_otef_:pit2-mCherry]ip^s^* ∆*spp1* | this study |
| SG200P_otef_:pep1-mCherry | *a1:mfa2 bE1 bW2  ip^r^[P_otef_:pep1-mCherry]ip^s^* | this study |
| SG200∆*spp1-*P_otef_:pep1-mCherry | *a1:mfa2 bE1 bW2* ∆*spp1  ip^r^[P_otef_:pep1-mCherry]ip^s^* | this study |
| SG200P_otef_:tin2-mCherry | *a1:mfa2 bE1 bW2  ip^r^[P_otef_:*tin2*-mCherry]ip^s^* | this study |
| SG200∆*spp1-*P_otef_:tin2-mCherry | *a1:mfa2 bE1 bW2* ∆*spp1  ip^r^[P_otef_:*tin2*-mCherry]ip^s^* | this study |
| SG200P_otef_:cmu1-mCherry | *a1:mfa2 bE1 bW2  ip^r^[P_otef_:*cmu1*-mCherry]ip^s^* | this study |
| SG200∆*spp1-*P_otef_:cmu1-mCherry | *a1:mfa2 bE1 bW2* ∆*spp1  ip^r^[P_otef_:*cmu1*-mCherry]ip^s^* | this study |

**(1) Scherer, M., Heimel, K., Starke, V., and Kämper, J. (2006).** The Clp1 protein is required for clamp formation and pathogenic development of *Ustilago maydis*. *The Plant Cell* **18**, 2388-2401
**(2) Heimel, K., Freitag, J., Hampel, M., Ast, J., Bölker, M., Kämper, J. (2013).** Crosstalk between the unfolded protein response and pathways that regulate pathogenic development in *Ustilago maydis*. *The Plant Cell* **25**, 4262–4277 **(3) Kämper, J., Kahmann, R., Bölker, M., Ma, L.J., Brefort, T., Saville, B.J., Banuett, F., Kronstad, J.W., Gold, S.E., et al. (2006).** Insights from the genome of the biotrophic fungal plant pathogen *Ustilago maydis*. *Nature* **444**, 97-101
**(4) Heimel, K., Scherer, M., Schuler, D., and Kämper, J. (2010a).** The *Ustilago maydis* Clp1 protein orchestrates pheromone and b-dependent signaling pathways to coordinate the cell cycle and pathogenic development. *The Plant Cell* **22**, 2908-2922
**(5) Hampel, M., Jakobi, M., Schmitz, L., Meyer, U., Finkernagel, F., Doehlemann, G., Heimel, K., (2016).** Unfolded Protein Response (UPR) Regulator Cib1 Controls Expression of Genes Encoding Secreted Virulence Factors in *Ustilago maydis*. *PloS one* **11**
